# GSARC: a group shuffle activated redundant connection framework for post-hoc feature space adaptation in skin lesion classification on the HAM10000 dataset

**DOI:** 10.3389/frai.2026.1867177

**Published:** 2026-08-18

**Authors:** Kumar Abhishek, Muralitharan Aishwaryaa Shree, Vinesh Kannaa Balaji, Rathna R

**Affiliations:** School of Computer Science and Engineering (SCOPE), Vellore Institute of Technology, Chennai, India

**Keywords:** skin lesion classification, deep convolutional neural network (DCNN), post-hoc adaptation, feature space adaptation, GSARC, HAM10000

## Abstract

**Introduction:**

Deep convolutional neural networks learn rich feature representations; however, the final classification head may not fully align with this feature space after end-to-end training, leading to underutilization of discriminative information. To address this limitation, we propose a lightweight post-hoc redundant head mechanism that improves feature-space adaptation without modifying or retraining the backbone network.

**Methods:**

The proposed approach introduces an additional shallow classification head trained from scratch on frozen feature representations augmented with frozen class logits. This design preserves training stability while enabling broader learning-rate exploration within an expanded signal space. The inclusion of frozen logits provides class-aware priors that stabilize optimization and support improved adaptation within the frozen feature manifold, allowing recovery of residual discriminative information with negligible computational overhead. The method was evaluated using a DenseNet-121 backbone enhanced with a Group–Squeeze-Excitation–Shuffle (GSSE) feature block on the HAM10000 skin lesion dataset through seven-class training and clinically relevant binary evaluation.

**Results:**

The proposed method improved binary classification accuracy from *91.06% to 92.42%* (+1.36 percentage points) and macro-averaged F1-score from *0.8588 to 0.8748* (+0.0160). It also outperformed the strongest baseline accuracy of *91.11%* by *1.31 percentage points*, while introducing only negligible computational overhead.

**Discussion:**

These findings demonstrate that lightweight post-hoc redundant head learning with logit-aware feature augmentation provides a stable, computationally efficient, and practical strategy for enhancing feature-space adaptation and improving medical image classification performance without requiring modification or retraining of the backbone network.

## 1 Introduction

Skin cancer is one of the most prevalent forms of cancer worldwide, making early and accurate diagnosis essential for effective treatment and improved patient outcomes. In recent years, deep learning–based approaches have demonstrated strong performance in automated skin lesion classification using dermoscopic images. Convolutional neural networks (CNNs), including DenseNet, EfficientNet, and Xception-based architectures, achieve high accuracy by learning hierarchical and discriminative visual representations from large-scale datasets such as HAM10000 (Calderón et al., 2021; Singh et al., 2022; Ali et al., 2022; Tschandl et al., 2018).

To further improve performance, prior studies have explored architectural enhancements, ensemble learning, transfer learning, attention mechanisms, and feature fusion strategies (Rahman et al., 2021; Salma and Eltrass, 2022; Ma et al., 2023; Salamaa and Aly, 2021; Mobiny et al., 2019; Roge et al., 2023). In addition, lightweight models have been proposed to support deployment in resource-constrained and clinical environments (Huang et al., 2021; Srinivasu et al., 2021). However, most existing approaches rely on end-to-end retraining or architectural modifications, which increase computational cost and reduce flexibility in post-deployment settings. Although modern CNN architectures can learn rich and expressive feature representations, they implicitly assume that the final classification head fully adapts to the learned feature space during end-to-end training. In practice, this assumption may not always hold, particularly when feature enrichment mechanisms such as channel attention, grouped convolutions, or feature shuffling are introduced (Zhang et al., 2018; Wang et al., 2018; Ramamurthy et al., 2024). These mechanisms expand the representational capacity of the feature space, but the classification head may not fully exploit the available discriminative information, which can manifest as suboptimal decision boundaries or increased confusion between visually similar classes.

As a result, part of the discriminative signal embedded in the feature space, along with class-specific decision information captured at the classifier level, can remain underutilized. Existing solutions attempt to address this limitation through full network retraining, ensemble methods, or additional supervision, which increase training complexity and computational overhead (Ma et al., 2023; Bechelli and Delhommelle, 2022; Akilandasowmya et al., 2024). Consequently, there is a lack of lightweight post-training approaches that can adapt classification behavior–such as improving class separation or reducing misclassification–within an already learned feature space without modifying or retraining the backbone network.

This limitation is particularly relevant in medical imaging pipelines, where retraining may be impractical due to computational, regulatory, or deployment constraints. To address this, we propose a lightweight post-hoc learning mechanism that improves feature space adaptation without modifying or retraining the backbone network. The approach introduces an additional shallow classification head trained on frozen feature representations augmented with frozen output logits, enabling stable refinement of decision boundaries. This mechanism is instantiated through the GSARC framework, built on a DenseNet-121 backbone enhanced with a Group–Squeeze-Excitation–Shuffle (GSSE) feature block that exposes richer yet underutilized representations. The proposed method enables effective exploitation of this enriched feature space while preserving the original model parameters. Its effectiveness is validated on the HAM10000 dataset under a clinically relevant binary setting, demonstrating consistent improvements in accuracy and macro-averaged F1-score with negligible computational overhead.

## 2 Related work

Recent research in automated skin lesion classification has primarily focused on improving diagnostic accuracy using deep learning models trained on dermoscopic images. Early approaches relied on conventional convolutional neural networks (CNNs) and transfer learning to address challenges such as limited labeled data and class imbalance (Srinivasu et al., 2021; Shetty et al., 2022; Fraiwan and Faouri, 2022; Chaturvedi et al., 2020; Jain et al., 2021; Ameri, 2020). Building on this foundation, more recent studies have explored architectural improvements. For instance, bilinear CNNs were introduced to better capture fine-grained feature interactions (Calderón et al., 2021), while modifications to Xception (Mehmood et al., 2023) and EfficientNet-based models (Ali et al., 2022) have focused on improving representation efficiency and balancing accuracy with computational cost.

In addition to architectural design, several works have incorporated ensemble learning, feature fusion, and hybrid classification strategies to improve robustness and generalization (Rahman et al., 2021; Ma et al., 2023; Roge et al., 2023; Akilandasowmya et al., 2024). Attention mechanisms have been used to emphasize clinically relevant regions, while uncertainty estimation and explainability techniques aim to improve model reliability and interpretability in clinical settings (Singh et al., 2022; Mobiny et al., 2019). More recently, transformer-based models and non-local operations have been explored to capture long-range dependencies and further expand representational capacity (Wang et al., 2018; Himel et al., 2024).

Lightweight and deployment-oriented architectures have also been proposed to support real-time inference in resource-constrained clinical environments (Huang et al., 2021; Zhang et al., 2018). The development and evaluation of these approaches have been largely driven by public benchmarks such as the HAM10000 dataset (Tschandl et al., 2018), which is often combined with data augmentation strategies to mitigate class imbalance and improve robustness (Shorten and Khoshgoftaar, 2019). Additional studies have explored a range of machine learning and deep learning frameworks, including hybrid CNN-based classifiers and optimized training pipelines, to improve diagnostic performance across diverse lesion categories (Tajerian et al., 2023; Mehr and Ameri, 2022; Ali et al., 2021; Zhang et al., 2024).

Despite these advancements, most existing approaches rely on end-to-end training or architectural modifications to achieve performance improvements. This dependence increases computational cost and limits flexibility in post-deployment settings. Furthermore, current methods provide limited support for adapting model behavior after training, which can restrict their applicability in clinical environments where retraining may be impractical.

Another important limitation is the implicit assumption that the final classification head fully adapts to the learned feature space during training. While feature enhancement techniques can produce richer and more expressive representations, existing approaches do not explicitly address potential misalignment between these features and the classification layer. As a result, some discriminative information may remain underutilized, particularly in cases involving visually similar classes.

Furthermore, there is a lack of lightweight post-training approaches that can improve classification behavior without modifying or retraining the backbone network. Existing refinement strategies are often limited to calibration methods or require additional training complexity, which reduces their practicality in real-world deployment scenarios.

These limitations highlight the need for efficient post-hoc methods that can better utilize learned feature representations while maintaining computational efficiency and deployment flexibility. While recent studies have primarily focused on improving skin lesion classification through architectural modifications, ensemble learning, attention mechanisms, or extensive retraining procedures, comparatively less attention has been given to lightweight post-training adaptation strategies that operate on frozen feature representations. The proposed GSARC framework addresses this gap by investigating whether additional discriminative information can be recovered from an already learned feature space through a computationally efficient post-hoc adaptation mechanism.

## 3 Proposed methodology

### 3.1 Base model

**Figure 1:**
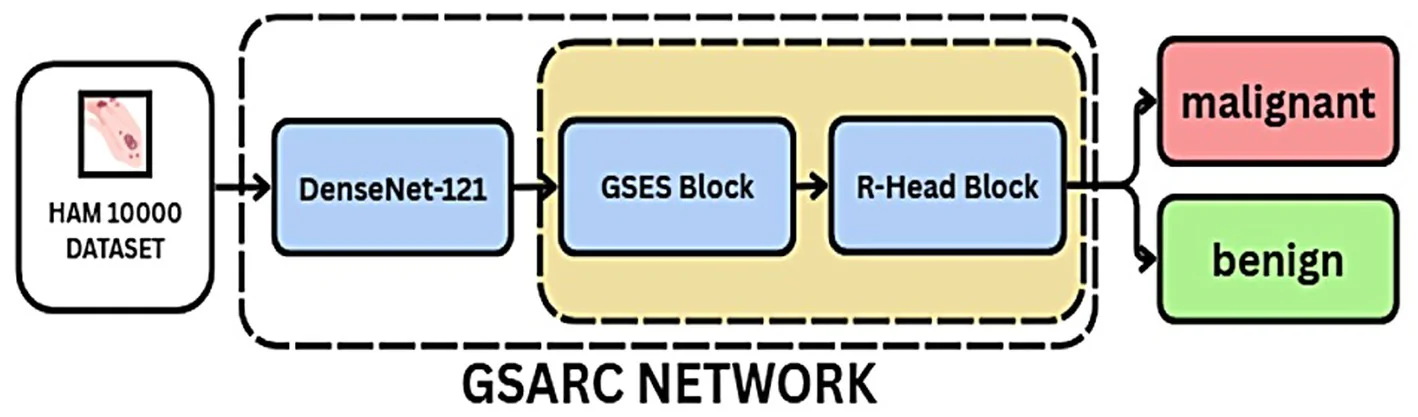
Simplified GSARC model diagram.

The base model is constructed using a DenseNet-121 architecture pretrained on ImageNet. DenseNet-121 is selected due to its strong feature reuse capability and proven effectiveness in medical image classification tasks. The original classification layer is replaced with a fully connected layer that produces seven output logits corresponding to the HAM10000 lesion categories. The network is trained end-to-end using cross-entropy loss and the Adam optimizer under a controlled hyperparameter setting to ensure stable convergence and fair comparison (Figure 1).

A minimal learning-rate search is performed using two candidate values (3 × 10^−4^ and 1 × 10^−4^). The model is trained for 50 epochs, during which performance is evaluated at each epoch to monitor convergence behavior. The best-performing checkpoint within this training run is selected based on validation accuracy, with macro-averaged F1-score used as a secondary criterion.

Although training follows a seven-class formulation, predictions are converted to a clinically relevant binary setting (malignant vs. benign) for evaluation. This binary evaluation reflects clinically relevant decision-making, where distinguishing malignant from benign lesions is critical. The best baseline configuration is retained and used as the reference model for subsequent feature enhancement and post-hoc adaptation.

### 3.2 GSSE feature enhancement block

To address redundancy and noise in dermoscopic data—where visually similar lesions and repeated acquisition patterns can lead to highly correlated feature responses—a Group–Squeeze-Excitation–Shuffle (GSSE) block is introduced after the final dense block of the DenseNet-121 backbone.

The backbone feature extractor remains unchanged, and the GSSE block operates on high-level feature maps to improve feature discrimination. The block consists of three sequential operations.

The mathematical formulation of the proposed GSSE feature enhancement block is presented in Equations (1)–(3). First, grouped pointwise convolution partitions the channel space into multiple groups, encouraging localized feature specialization while reducing inter-channel correlation. For an input feature map *X* ∈ R^*C* × *H* × *W*^, this operation is defined as:

$$X_{g}={\text{Conv}}^{\left(g\right)}(X)$$ (1)

Where *g* denotes the number of channel groups.

Second, a squeeze-and-excitation module performs channel-wise recalibration using global context. This suppresses less informative channels while emphasizing discriminative responses:

$$s=\sigma \;(W_{2}\;\delta \;(W_{1}\;\mathrm{GAP}(X_{g}))),X^{ˆ}=X_{g}⊙s$$ (2)

Where GAP(·) denotes global average pooling, *W*_1_ and *W*_2_ are learned parameters, *δ* (·) and *σ* (·) represent ReLU and sigmoid activations, respectively, and ⊙ denotes channel-wise multiplication.

Finally, a channel shuffle operation redistributes features across groups to enable cross-group information exchange:

$$X_{\text{shuffled}}=\pi (X^{ˆ})$$ (3)

Where *π*(·) denotes channel permutation.

The GSSE block improves feature selectivity and robustness under redundant signal conditions. However, the primary classification head, optimized during end-to-end training, may not fully adapt to this enriched feature space. This limitation motivates the proposed post-hoc redundant head, which adapts classification behavior to the enriched feature space without altering learned representations.

### 3.3 Redundant post-hoc classification head

To address the misalignment between enriched feature representations and the primary classification head, a post-hoc redundant head (R-Head) is introduced.

The redundant head is an auxiliary classifier trained on frozen representations from the fully trained GSSE-enhanced model. The backbone and original classification head remain unchanged to preserve learned features.

The mathematical formulation of the proposed post-hoc redundant classification head is presented in Equations (4)–(7). Given an input image *x*, the frozen backbone produces a feature representation:

f=B(x) (4)

Where *B*(·) denotes the GSSE-enhanced feature extractor. The original classification head produces logits:

h=C(f) (5)

These logits are retained without modification. The redundant head is trained on a concatenation of feature embeddings and logits:

z=[fh] (6)

The final prediction is given by:

yˆ=R(z) (7)

Where *R*(·) represents a shallow linear classifier trained *post hoc*.

A linear classifier was intentionally selected for the redundant head to preserve the lightweight nature of the proposed framework. The objective of this work is not to increase classifier complexity, but rather to investigate whether improved post-hoc adaptation can recover additional discriminative information from an already learned feature space without modifying the backbone network. By retaining a simple linear classifier, performance improvements can be more directly attributed to the proposed adaptation mechanism and the utilization of frozen features and logits, rather than to increased model capacity.

Furthermore, a shallow architecture minimizes computational overhead, reduces the risk of overfitting on frozen feature representations, and enables efficient deployment and rapid optimization. More expressive post-hoc classifiers, such as multilayer perceptrons or regularized nonlinear architectures, may provide additional modeling capacity and potentially further improve performance. However, such approaches introduce additional hyperparameter dependencies, tuning requirements, and computational complexity, which are contrary to the lightweight design philosophy of the proposed framework. Exploring more sophisticated redundant-head architectures while preserving computational efficiency represents an important direction for future work.

Incorporating frozen logits provides class-aware priors that stabilize optimization and guide learning within the augmented feature space, while GSSE features retain high-dimensional semantic information. This combination enables effective utilization of previously underexploited discriminative signals without altering learned representations.

Training is performed using learning rates selected from {1 × 10^−3^, 3 × 10^−3^, 1 × 10^−4^, 3 × 10^−4^}. The redundant head is trained for up to 30 epochs, with performance evaluated at each epoch. The best-performing checkpoint is selected based on validation performance.

This lightweight training strategy allows adaptation to the expanded signal space while maintaining minimal computational overhead.

### 3.4 Training and inference protocol

The overall pipeline separates feature learning from post-hoc adaptation to ensure stability and efficiency. The procedure consists of four stages.

#### 3.4.1 Stage 1 – base model training

The DenseNet-121 backbone is trained end-to-end using a seven-class formulation under controlled hyperparameter settings. Performance is monitored across epochs, and the best checkpoint is selected based on validation accuracy, with macro-averaged F1-score as a secondary metric.

To avoid ambiguity, all reported performance values correspond to evaluation on the held-out lesion-wise test partition. The same train-test split was maintained across all experiments to ensure fair comparison among model variants.

#### 3.4.2 Stage 2 – feature enhancement training

The GSSE block is integrated into the backbone, and the model is retrained under the same protocol. The best-performing checkpoint is selected based on validation accuracy.

#### 3.4.3 Stage 3 – redundant head training

**Figure 2:**
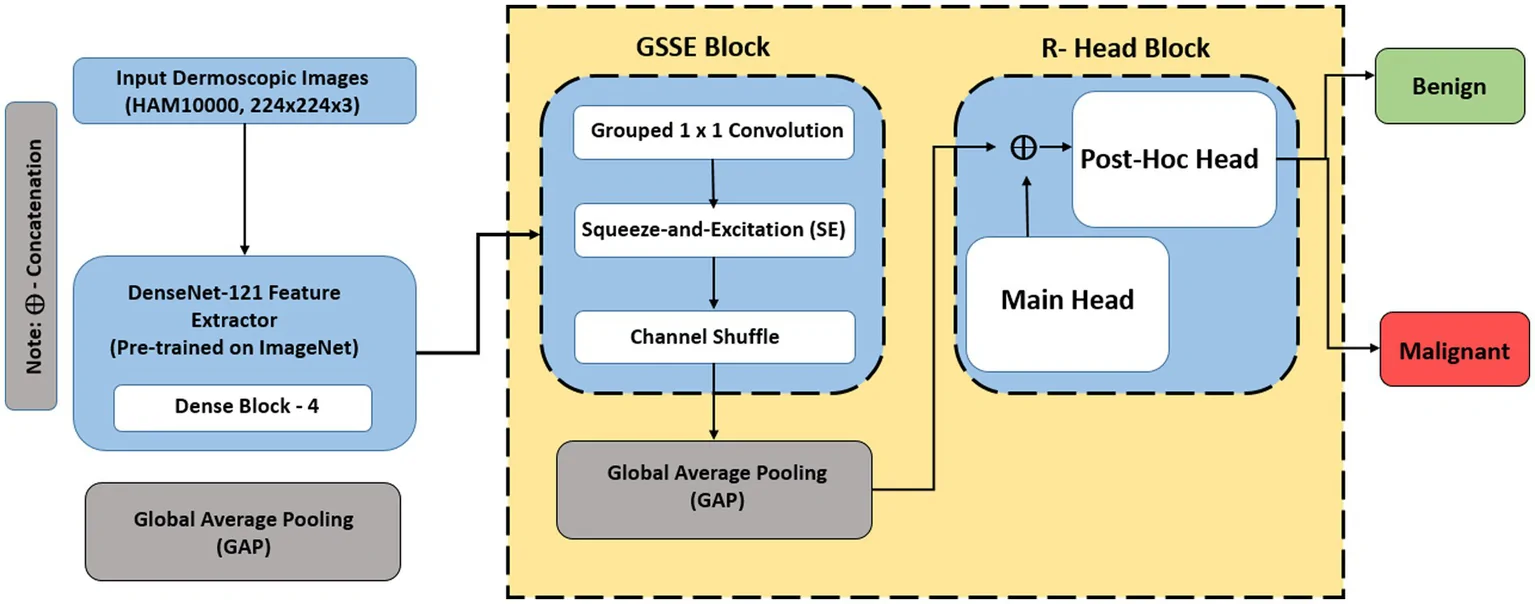
Detailed GSARC model diagram.

After convergence, the GSSE-enhanced backbone and its original classification head are frozen. The redundant head is trained from scratch on frozen representations using lightweight learning-rate exploration. The best checkpoint is selected based on validation performance (Figure 2).

#### 3.4.4 Stage 4 – inference

During inference, predictions are generated exclusively by the redundant head. The original classification head remains unchanged and is used only during training as a source of auxiliary information.

The final deployed model consists of the GSSE-enhanced backbone combined with the best-performing redundant head.

### 3.5 Dataset description

The experiments are conducted on the HAM10000 dataset (Human Against Machine with 10,000 training images), a widely used public benchmark for dermoscopic skin lesion classification. The dataset contains 10,015 dermoscopic images collected from multiple clinical sources using diverse imaging devices and acquisition settings, resulting in significant variability in resolution, illumination, color distribution, and lesion appearance that reflects realistic clinical conditions.

The dataset comprises seven diagnostic categories of pigmented skin lesions: melanoma (mel), melanocytic nevi (nv), basal cell carcinoma (bcc), actinic keratoses and intraepithelial carcinoma (akiec), benign keratosis-like lesions (bkl), dermatofibroma (df), and vascular lesions (vasc). These categories exhibit strong visual similarity and class imbalance, with melanocytic nevi representing the majority class and malignant categories being comparatively underrepresented.

Each image is associated with a lesion identifier, enabling tracking of multiple images from the same lesion. To prevent patient-level and lesion-level data leakage, a lesion-wise split is employed, ensuring that all images from a given lesion are assigned exclusively to either the training or testing set. Approximately 80% of lesions are used for training, while the remaining 20% are reserved for evaluation.

Although training is performed using a seven-class classification framework to preserve fine-grained lesion characteristics, evaluation is conducted using a clinically meaningful binary setting. In this formulation, lesions are grouped into malignant (mel, bcc, akiec) and benign (nv, bkl, df, vasc) categories, aligning the evaluation protocol with practical diagnostic screening scenarios.

### 3.6 Data preprocessing and augmentation

All dermoscopic images are resized to 224 × 224 pixels to match the input resolution required by the pretrained DenseNet-121 backbone. The images are normalized using ImageNet mean and standard deviation values to ensure compatibility with pretrained weights and to support stable optimization.

Dataset partitioning is performed using a lesion-wise splitting strategy, where lesion identifiers are randomly shuffled and approximately 80% are assigned to the training set and 20% to the testing set. Since the split is performed at the lesion level, all images corresponding to a given lesion are contained within a single partition, preventing patient-level and lesion-level data leakage.

During training, lightweight augmentation techniques are applied to improve generalization and reduce overfitting. These include random horizontal and vertical flips, which introduce spatial variability while preserving lesion semantics. No augmentation is applied during testing to ensure unbiased evaluation.

To address class imbalance, a weighted random sampling strategy is employed during training, where sampling probabilities are inversely proportional to class frequencies. This increases the representation of underrepresented classes without altering the dataset distribution or introducing artificial duplication.

All preprocessing steps, including lesion-wise splitting, augmentation, and sampling, are applied consistently across all experimental configurations to ensure fair comparison and reproducibility.

## 4 Results and analysis

### 4.1 Environmental setup and hyperparameter tuning

All experiments are conducted on the Kaggle platform using a dual NVIDIA T4 GPU configuration (T4 × 2). Reproducibility is ensured by fixing the random seed (SEED = 42) across Python’s random module, NumPy, and PyTorch. Deterministic computation is enforced by disabling cuDNN benchmarking and enabling deterministic behavior in PyTorch.

The detailed implementation settings provided in Table 1 are intended to improve reproducibility and facilitate independent verification of the reported results. All experiments were conducted using a fixed lesion-wise partition and explicitly reported hyperparameter configurations to ensure consistency across model variants.

**Table 1:** Detailed implementation configuration of the proposed GSARC framework.

Component	Configuration
Backbone	DenseNet-121 (ImageNet pretrained)
Input resolution	224 × 224
GSSE group count	4
GSSE convolution type	Grouped 1 × 1 Convolution
Squeeze-and-excitation reduction ratio	16
Channel shuffle	Enabled
Residual connections	Not used
Optimizer	Adam
Loss function	Cross-Entropy Loss
Batch size	32
Base model learning rates	{1 × 10^−4^, 3 × 10^−4^}
GSARC learning rates	{1 × 10^−4^, 3 × 10^−4^, 1 × 10^−3^, 3 × 10^−3^}
Base model training epochs	Up to 50
Redundant head training epochs	Up to 30
Random seed	42
Class imbalance strategy	Weighted Random Sampling
Backbone during GSARC training	Frozen
Original classifier during GSARC training	Frozen
Redundant head structure	Single Linear Layer
Redundant head input	Concatenated GSSE Features + Frozen Logits
Output classes	7

The redundant head operates on a concatenated representation consisting of pooled GSSE feature embeddings and the frozen logits generated by the original classifier. During GSARC training, all backbone parameters and the original classification head remain frozen, ensuring that only the lightweight redundant head is optimized.

This design preserves the learned feature space while enabling post-hoc adaptation with minimal computational overhead. The redundant head itself consists of a single linear classification layer trained using the Adam optimizer and cross-entropy loss. By restricting optimization to the redundant head, the proposed framework isolates the contribution of post-hoc adaptation from changes in backbone representations, thereby maintaining computational efficiency while recovering additional discriminative information from the existing feature space. Model training and hyperparameter exploration are performed in a unified and controlled setting. For baseline and feature-enhanced models, learning rates of 1 × 10^−4^ and 3 × 10^−4^ are explored within a fixed training horizon of up to 50 epochs. Model performance is evaluated at each epoch, and the best-performing checkpoint is selected based on validation accuracy, with macro-averaged F1-score used as a secondary criterion. The explored hyperparameters are summarized in Table 2.

**Table 2:** Hyperparameters for baseline and GSSE-enhanced training.

Hyperparameters	Values explored
Learning rates	1 × 10^−4^, 3 × 10^−4^
Epochs	Up to 50
Optimizer	Adam
Loss function	Cross-entropy
Batch size	32

For post-hoc redundant head training, the GSSE-enhanced backbone and its original classification head are fully frozen to preserve representational stability. The redundant head is trained from scratch using learning rates of 1 × 10^−4^ and 3 × 10^−4^ over a fixed horizon of up to 30 epochs. In the final GSARC configuration, extended learning-rate exploration (1 × 10^−3^ and 3 × 10^−3^) is introduced to better adapt to the enriched feature space. The best-performing configuration is reported in Table 3. During redundant head training, frozen output logits from the original classifier are concatenated with pooled GSSE feature representations. These logits act as stable class-aware priors, improving optimization stability and enabling more effective adaptation without modifying the backbone. The best-performing checkpoint is selected based on validation accuracy.

**Table 3:** Best hyperparameters for GSARC (base + R-Head).

Hyperparameters	Best configuration
Learning rates	3 × 10^−3^
Epoch	29

All models are optimized using the Adam optimizer with cross-entropy loss. A batch size of 32 is maintained across all experiments to ensure consistent comparison and efficient GPU utilization.

### 4.2 Performance analysis

The performance of the proposed GSARC framework is evaluated using binary classification accuracy and macro-averaged F1-score, which are appropriate for imbalanced medical datasets. All models are trained using a seven-class formulation and evaluated under a clinically relevant binary setting on a held-out lesion-wise test split. Best-performing checkpoints are selected based on validation performance within fixed training runs.

The baseline DenseNet-121 achieves a binary accuracy of 91.11%, establishing a strong reference point. Applying post-hoc redundant head training directly to this baseline yields only a marginal improvement (approximately 0.1% absolute gain), indicating limited residual discriminative capacity when the feature space is not explicitly enriched.

To address this limitation, a lightweight grouped convolution and channel shuffle mechanism is introduced after the final dense block. This modification improves the base accuracy to 91.16%, and subsequent post-hoc adaptation increases performance to 91.26%, suggesting partial utilization of previously underexplored discriminative features. However, the overall improvement remains modest.

In contrast, the full GSSE feature enhancement significantly enriches the representational space. The GSSE-enhanced model, without post-hoc adaptation, achieves a binary accuracy of 91.06% and a macro-averaged F1-score of 0.8588, indicating that the jointly trained primary classification head does not fully exploit the enriched feature representations.

When the proposed post-hoc redundant head is applied to the GSSE-enhanced backbone, a clear and consistent improvement is observed. The redundant head operates on a combination of pooled GSSE features and frozen logits from the primary classifier, where the logits provide stable class-aware priors that guide optimization.

The final GSARC model achieves a binary accuracy of 92.42% and a macro-averaged F1-score of 0.8748. This corresponds to an improvement of 1.36 percentage points in accuracy over the GSSE base model and surpasses the strongest baseline performance of 91.11%. The macro-F1 score also improves from 0.8588 to 0.8748 (+0.0160).

A comparison of all evaluated models is presented in Table 4. These results demonstrate that post-hoc redundant head learning becomes increasingly effective as feature enrichment exposes additional discriminative capacity, validating the design of GSARC and highlighting the importance of combining feature-space enhancement with logit-aware post-training adaptation (Table 5).

**Table 4:** Ablation models performance comparison.

Final model names (each experiment)	Binary accuracy	Macro avg F1
Baseline pretrained DenseNet-121	0.9111	0.8592
Enhanced baseline with group shuffle and R Head	0.9126	0.8540
Final proposed model	0.9242	0.8748

**Table 5:** Binary medical performance metrics of the proposed GSARC framework on the HAM10000 test set.

Metric	Formula	Value (%)
Sensitivity (recall/true positive rate)	TP/(TP + FN)	77.31
Specificity (true negative rate)	TN/(TN + FP)	96.00
False negative rate (FNR)	FN/(FN + TP)	22.69

#### 4.2.1 Clinical evaluation metrics

While overall accuracy and F1-score provide useful measures of classification performance, additional clinical metrics are necessary to assess the practical utility of the proposed framework in medical screening scenarios. In particular, sensitivity, specificity, and false negative rate (FNR) provide insight into the model’s ability to correctly identify malignant lesions while minimizing missed diagnoses.

The confusion matrix components corresponding to the proposed GSARC framework were: TP = 293, TN = 1,537, FP = 64, and FN = 86. From a clinical evaluation perspective, the model achieved a sensitivity of 77.31% and a specificity of 96.00%, corresponding to a false negative rate of 22.69%.

The high specificity demonstrates a strong capability to correctly identify benign lesions while minimizing unnecessary false-positive predictions and follow-up examinations. At the same time, the achieved sensitivity indicates that the majority of malignant lesions are successfully detected. Although a subset of malignant cases remained undetected, resulting in a moderate false negative rate, the proposed framework maintains a favorable balance between lesion detection and false-positive control. These findings suggest that GSARC may serve as a useful clinical decision-support tool, where both diagnostic reliability and reduction of unnecessary interventions are important considerations.

#### 4.2.2 Threshold-independent performance analysis

To further evaluate the discriminative capability of the proposed framework independent of any specific decision threshold, Receiver Operating Characteristic (ROC) and Precision-Recall (PR) analyses were performed. Since the original checkpoint used for the reported binary classification results was no longer available, these analyses were conducted using a reproduction run executed under the same experimental protocol. Although exact numerical replication of the previously reported accuracy was not obtained, the reproduced model exhibited comparable performance and was used solely to provide threshold-independent evaluation metrics requested by the reviewers.

**Figure 3:**
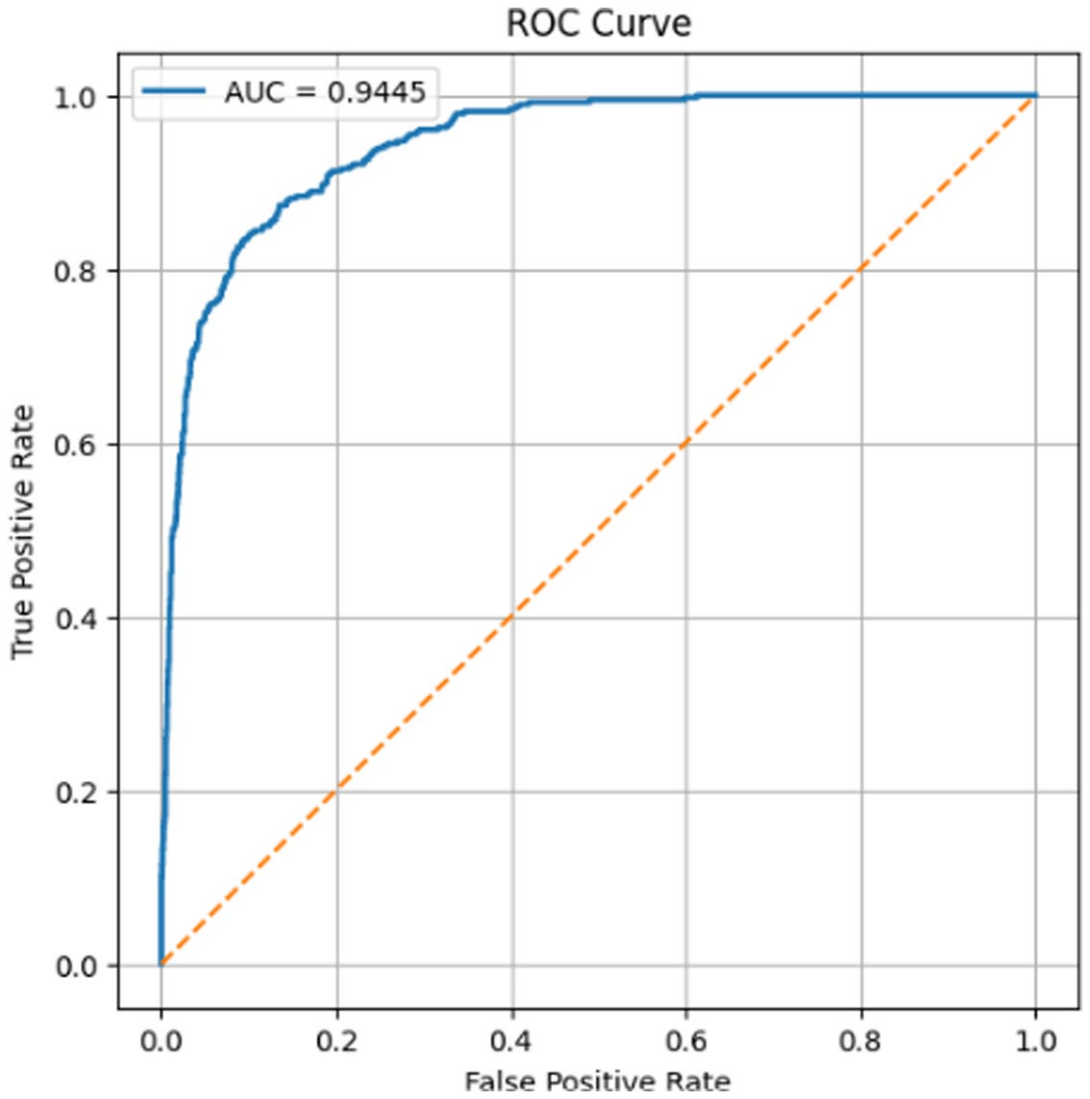
Receiver operating characteristic (ROC) curve of the reproduced GSARC framework.

The model achieved an AUC-ROC of 0.9445, indicating excellent discriminative capability between malignant and benign lesion classes across a wide range of decision thresholds. The ROC analysis yielded an AUC-ROC of 0.9445. As illustrated in Figure 3, the ROC curve remains substantially above the random-classification baseline across all operating thresholds. This behavior indicates strong separation between malignant and benign lesion classes and demonstrates that the proposed framework can maintain high true-positive rates while effectively controlling false-positive predictions. The high AUC-ROC value suggests excellent discriminative capability and supports the suitability of GSARC for clinical screening applications.

The model achieved a PR-AUC of 0.8221, demonstrating strong precision-recall trade-offs under class imbalance. Given the class imbalance present in the HAM10000 dataset, Precision-Recall analysis was also performed. The proposed framework achieved a PR-AUC of 0.8221, indicating strong performance in maintaining precision while increasing recall.

**Figure 4:**
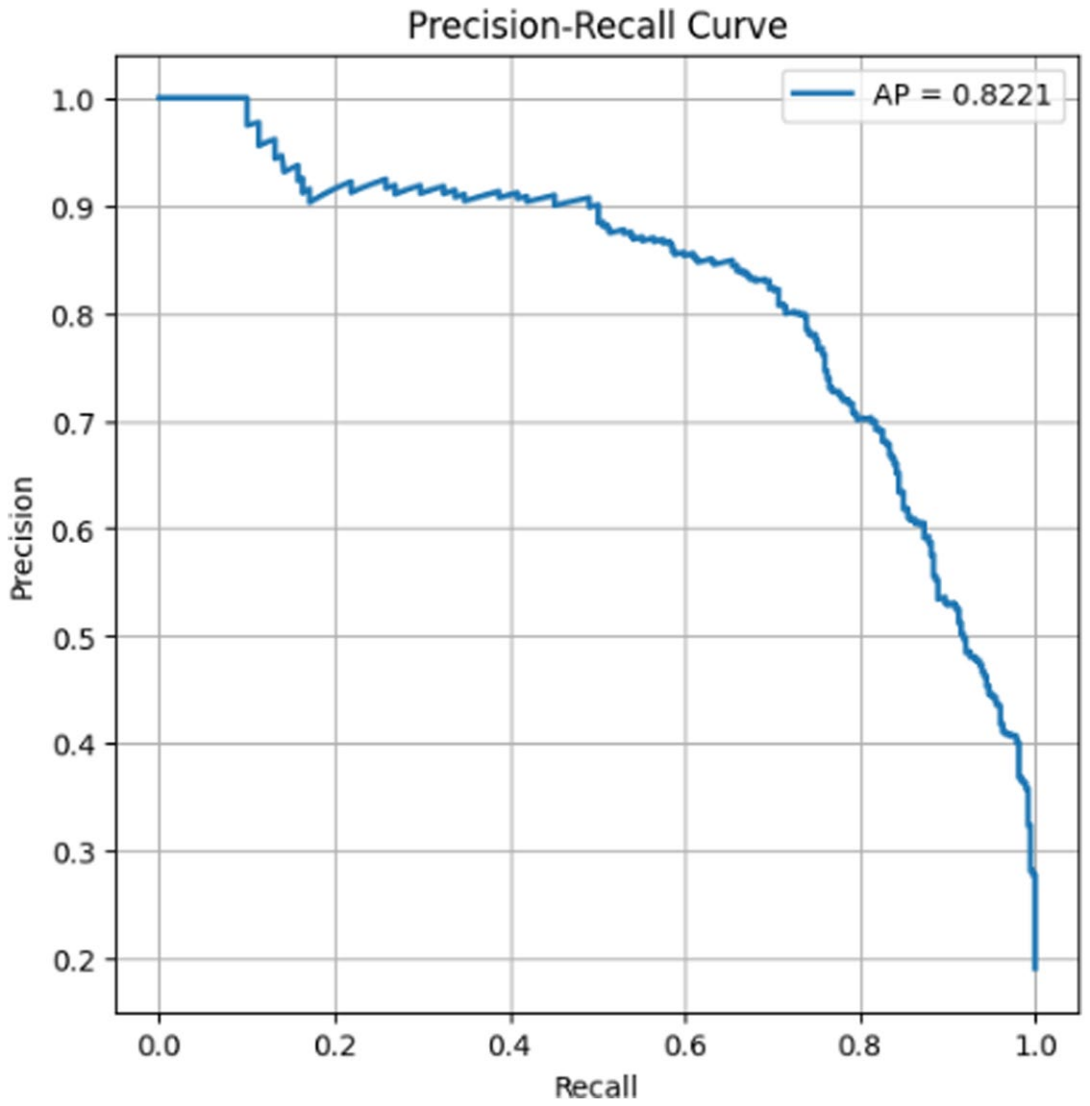
Precision-recall (PR) curve of the reproduced GSARC framework.

As shown in Figure 4, precision remains high across a substantial portion of the recall range, suggesting that the model can identify malignant lesions effectively without generating excessive false-positive predictions. Since Precision-Recall analysis is particularly informative under imbalanced class distributions, the strong PR-AUC further supports the robustness of the proposed framework for skin lesion classification tasks where malignant cases are comparatively less frequent.

Collectively, the sensitivity, specificity, FNR, ROC-AUC, and PR-AUC results provide a more comprehensive clinical assessment of the proposed GSARC framework. The combination of high specificity, strong discriminative capability, and favorable precision-recall characteristics indicates that the proposed lightweight post-hoc adaptation mechanism improves not only overall classification performance but also clinically relevant diagnostic behavior.

### 4.3 Ablation studies

All ablation experiments are conducted using an identical preprocessing and evaluation pipeline to ensure fair comparison. Images are resized to 224 × 224 pixels and normalized using ImageNet statistics. A lesion-wise 80/20 train–test split is employed to prevent data leakage, ensuring that images of the same lesion do not appear across splits.

During training, random horizontal and vertical flips are applied, and class imbalance is addressed using weighted random sampling. No augmentation is applied during testing. Random seeds are fixed (SEED = 42), and all experiments are performed under identical conditions.

#### 4.3.1 Experiment 1: baseline evaluation

In the first experiment, a pretrained DenseNet-121 backbone is evaluated as the baseline model. The network is trained end-to-end using a seven-class formulation, while evaluation is performed under a binary malignant–benign setting. Training and hyperparameter exploration are conducted jointly, with learning rates selected from {1 × 10^−4^, 3 × 10^−4^}.

The model is trained for up to 50 epochs, with performance evaluated at each epoch to monitor convergence. The best-performing checkpoint is selected based on binary accuracy, with macro-averaged F1-score used as a secondary criterion.

**Figure 5:**
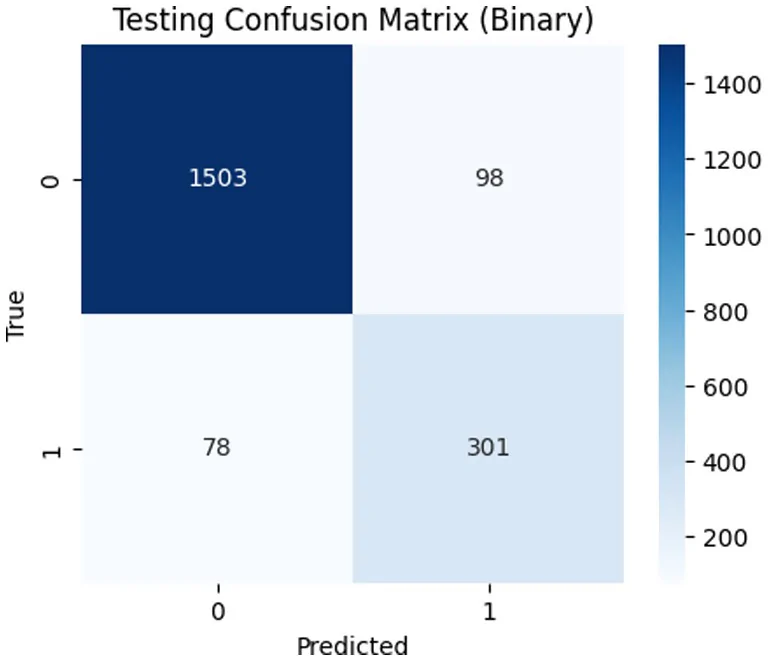
Baseline model confusion matrix.

**Figure 6:**
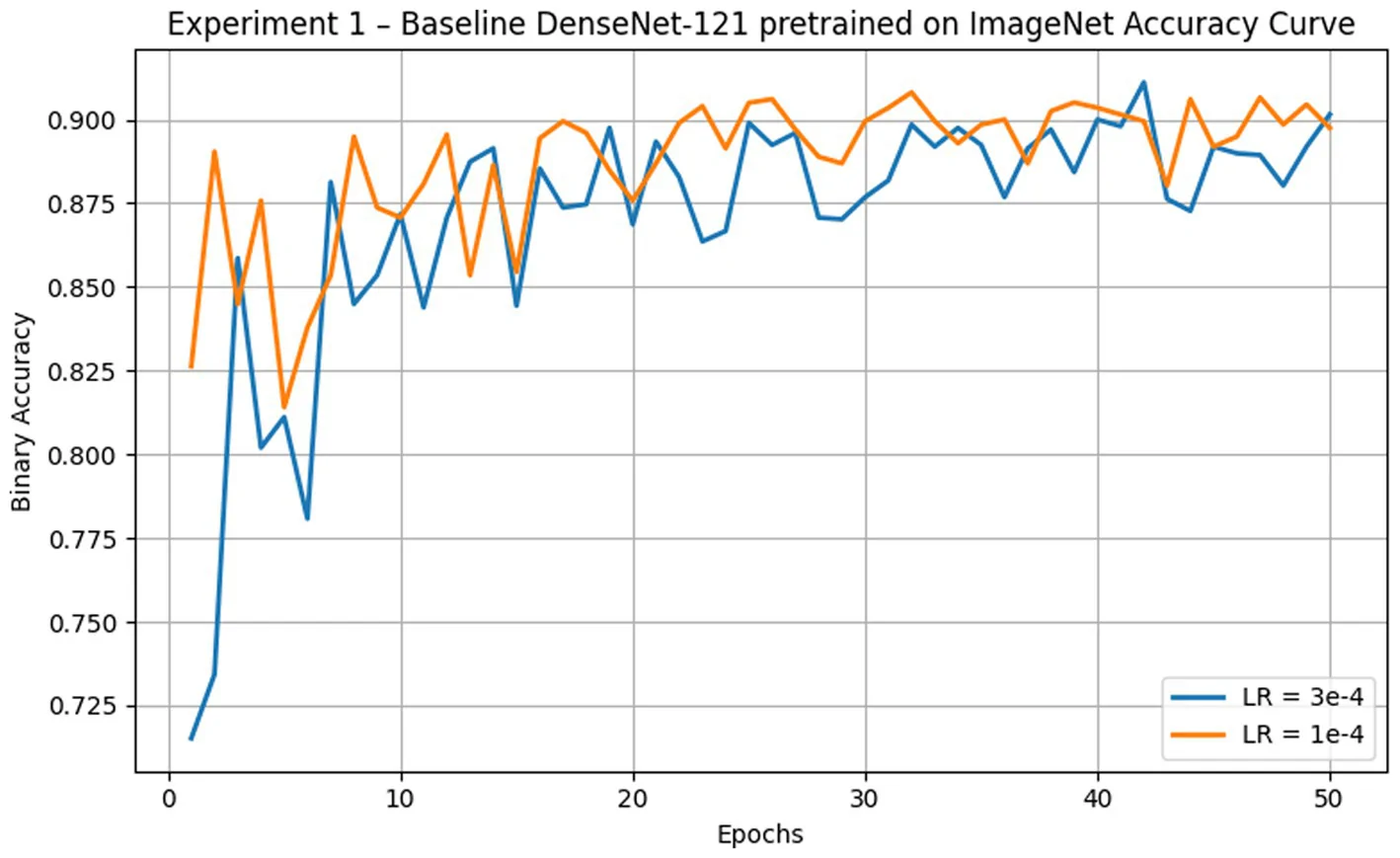
Baseline model training-tuning binary accuracy curve.

**Figure 7:**
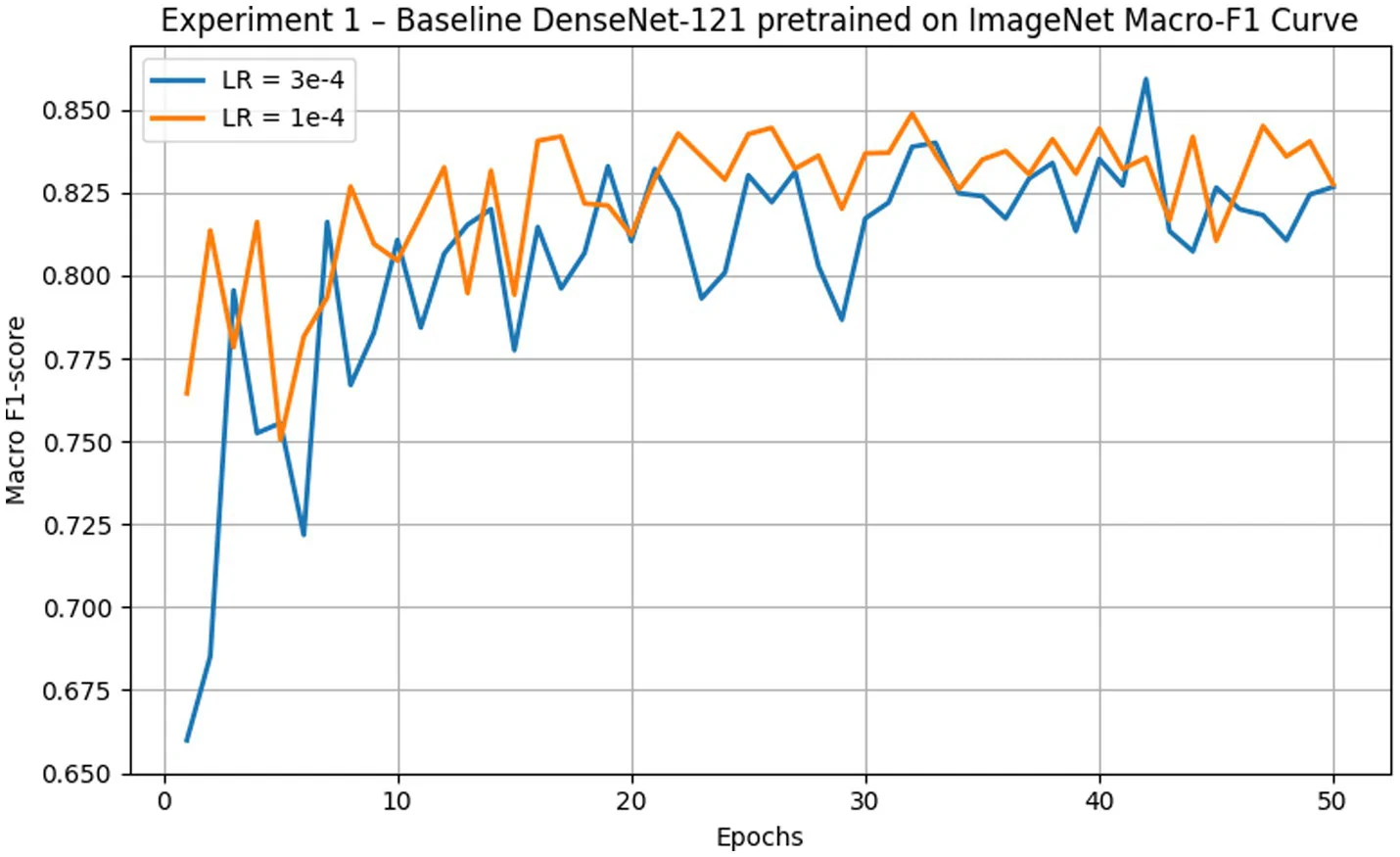
Baseline model training-tuning macro avg F1 curve.

The best baseline configuration achieves a binary accuracy of 91.11% and a macro-averaged F1-score of 0.8592 on the lesion-wise test set. As shown in the classification report (Table 6), the model achieves balanced performance across classes under the binary setting. The confusion matrix (Figure 5) indicates strong performance on the benign class, while misclassifications are higher for malignant cases, reflecting dataset imbalance. The training curves (Figures 6, 7) show stable convergence without significant overfitting.

**Table 6:** Baseline model performance classification report.

Class	Precision	Recall	F1-score	Support
0	0.9507	0.9388	0.9447	1,601
1	0.7544	0.7942	0.7738	379
Accuracy		0.9111		1,980
Macro avg	0.8525	0.8665	0.8592	1,980
Weighted avg	0.9131	0.9111	0.9120	1,980

DenseNet-121 is selected due to its dense connectivity, which promotes feature reuse and preserves fine-grained spatial information. These properties are particularly beneficial for dermoscopic analysis, where subtle variations in texture and color are diagnostically important. Additionally, it provides a balance between representational capacity and computational efficiency, making it suitable for evaluating post-hoc feature utilization.

#### 4.3.2 Experiment 2: baseline + group-shuffle + redundant head

##### 4.3.2.1 Base model training

In the second experiment, the DenseNet-121 backbone is augmented with a lightweight grouped convolution and channel shuffle head applied after the final dense block. This design excludes squeeze-excitation and residual connections and aims to increase channel-wise feature diversity under the same training protocol.

Training is conducted using learning rates {1 × 10^−4^, 3 × 10^−4^} for up to 50 epochs. Model selection follows the same criteria as Experiment 1.

**Figure 8:**
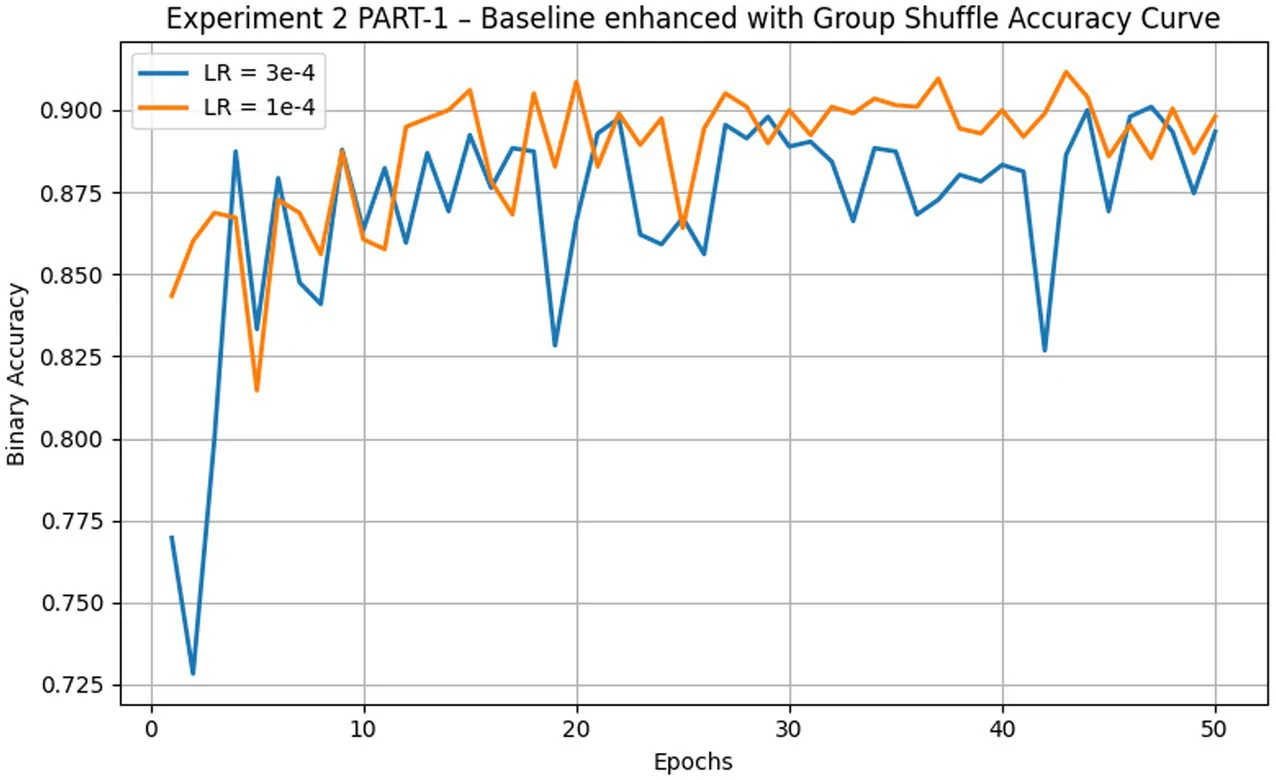
Baseline model + group shuffle training-tuning binary accuracy curve.

**Figure 9:**
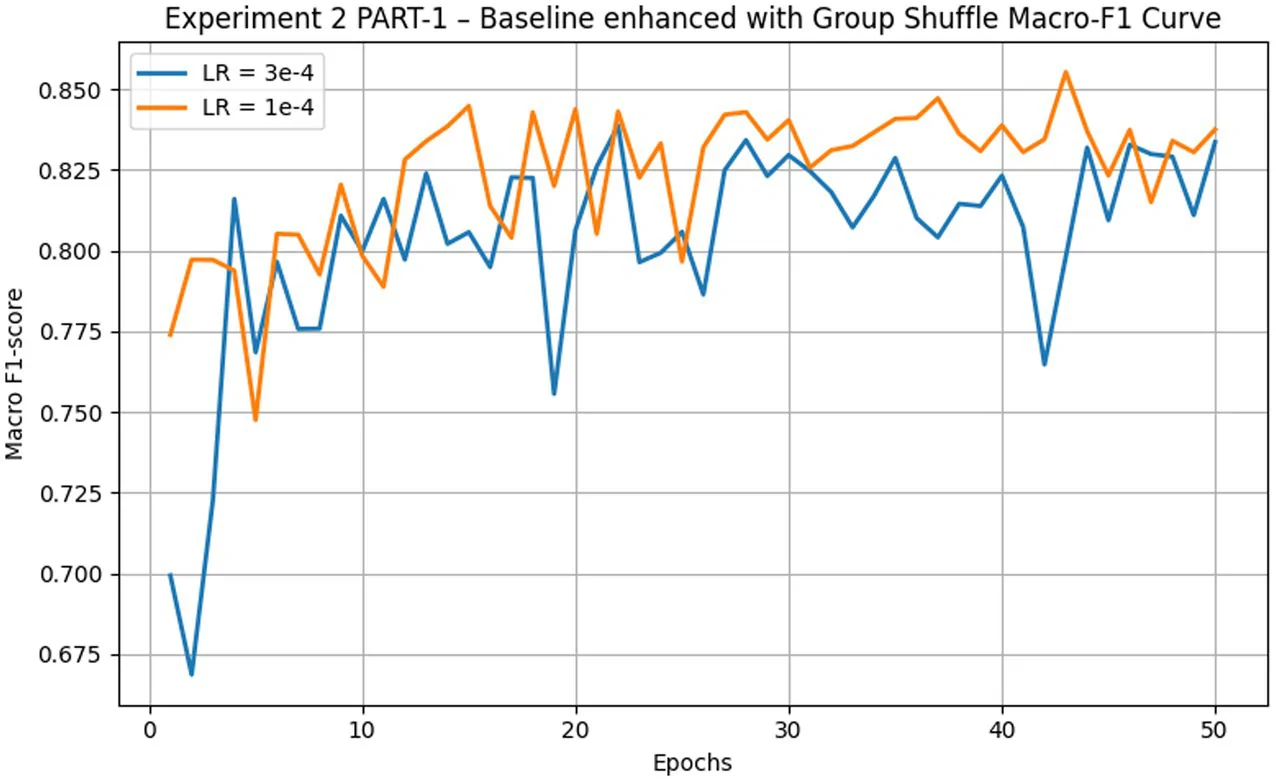
Baseline model + group-shuffle training-tuning macro avg F1 curve.

The best-performing configuration achieves a binary accuracy of 91.16% and a macro-averaged F1-score of approximately 0.85. As shown in Table 7, the classification performance improves slightly over the baseline. The training curves (Figures 8, 9) indicate stable convergence similar to the baseline model.

The modest improvement suggests that while channel grouping and shuffle operations increase feature diversity, they do not sufficiently structure inter-channel relationships for effective downstream classification. As a result, the primary classification head is unable to fully utilize the modified feature space (Table 7).

**Table 7:** Baseline model + group-shuffle performance classification report.

Class	Precision	Recall	F1-score	Support
0	0.9418	0.9494	0.9456	1,601
1	0.7787	0.7520	0.7651	379
Accuracy		0.9116		1,980
Macro avg	0.8602	0.8507	0.8553	1,980
Weighted avg	0.9105	0.9116	0.9110	1,980

##### 4.3.2.2 Redundant head training on frozen backbone

To further evaluate feature utilization, a redundant head (R-Head) is trained on the frozen group-shuffle–enhanced backbone. All backbone parameters, including the original classification head, are frozen. The redundant head is trained from scratch using frozen representations.

Training is performed for up to 30 epochs with learning rates {1 × 10^−4^, 3 × 10^−4^}, and model selection follows the same criteria.

**Figure 10:**
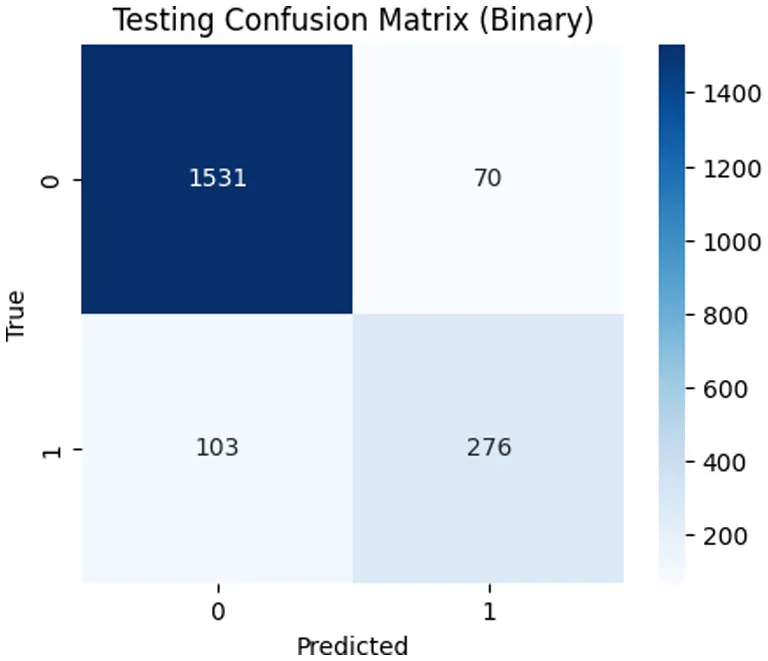
Baseline model + group-shuffle + R-Head confusion matrix.

**Figure 11:**
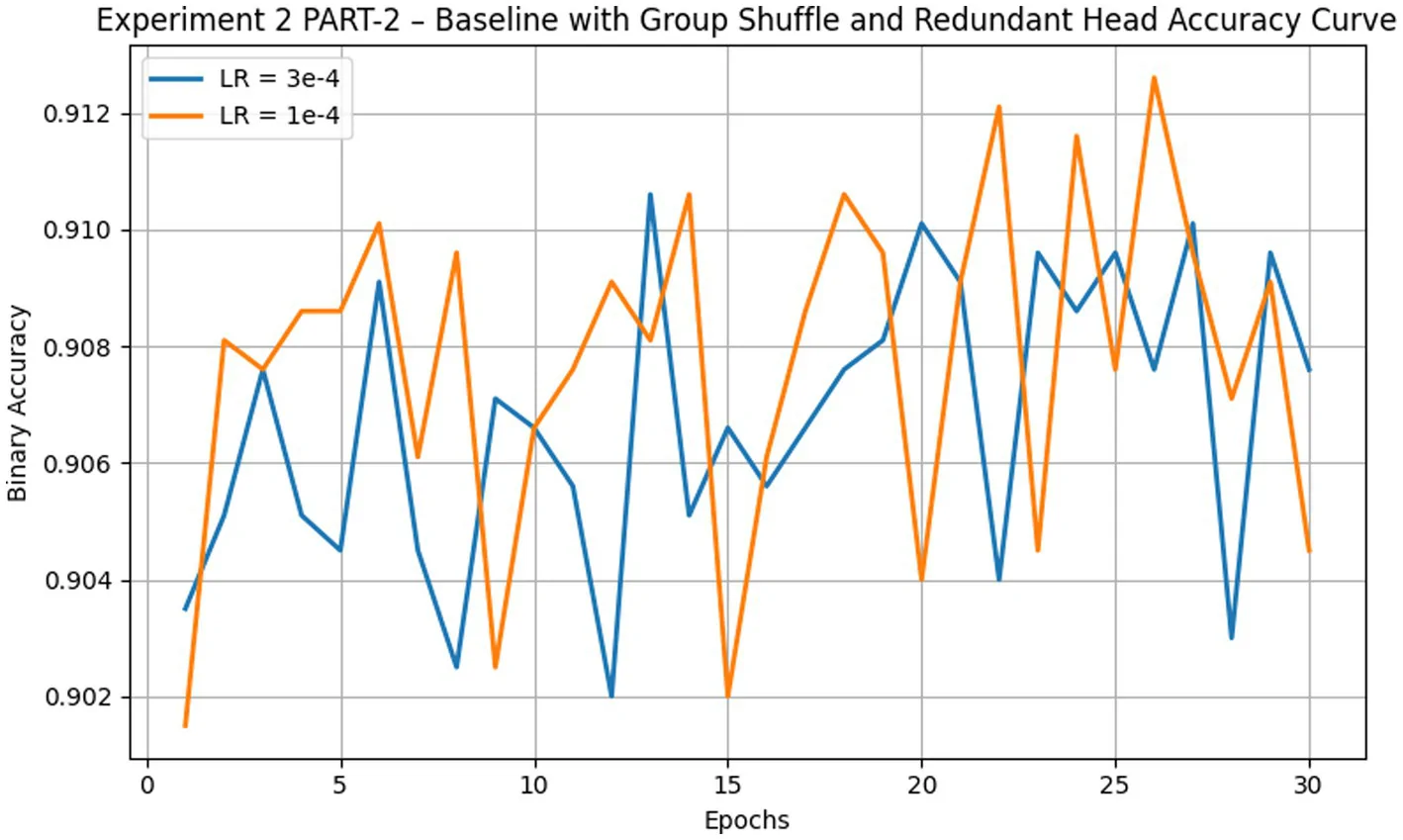
Baseline model + group-shuffle + R-Head training-tuning binary accuracy curve.

**Figure 12:**
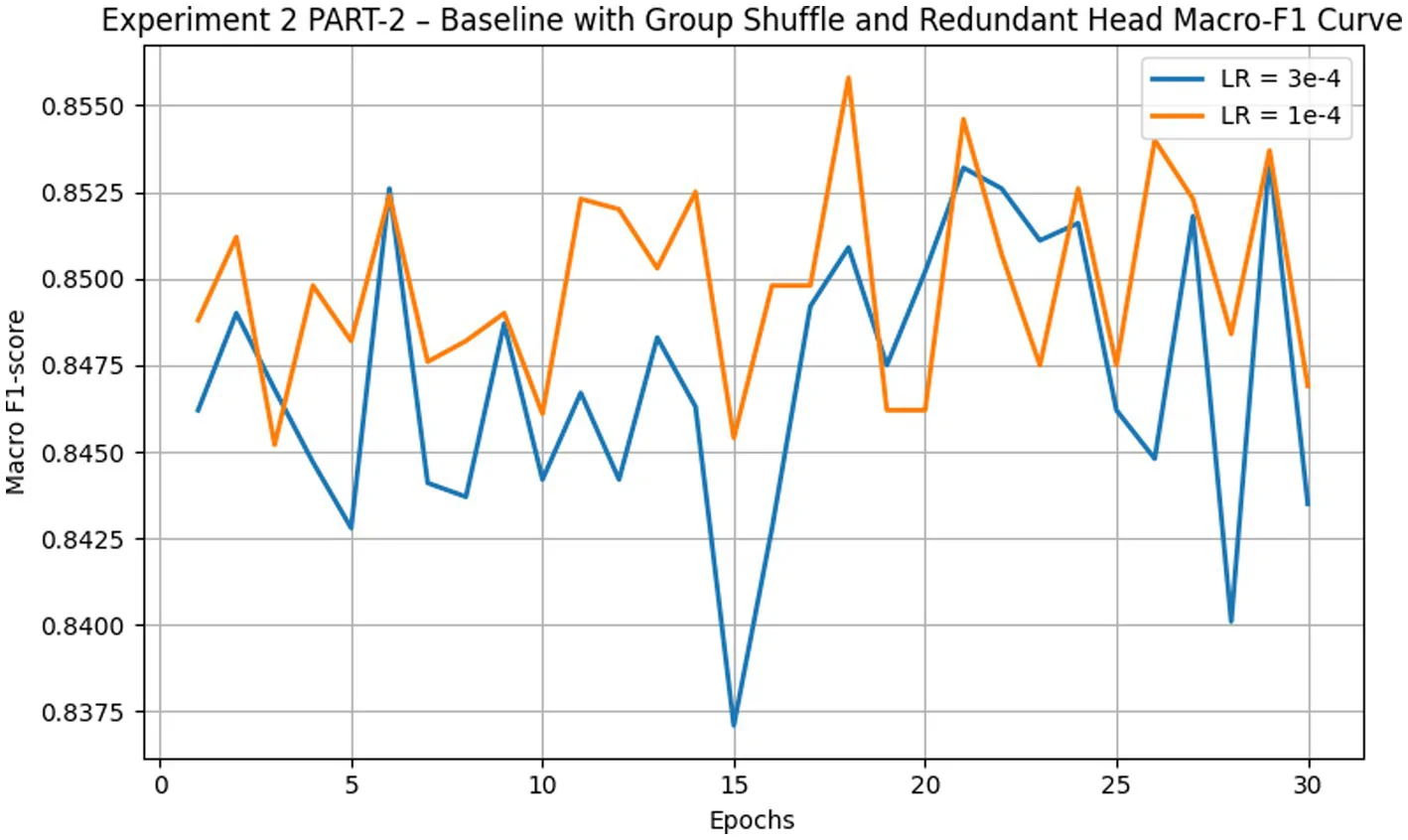
Baseline model + group-shuffle + R-Head training-tuning macro avg F1 curve.

This configuration achieves a binary accuracy of 91.26% with a macro-averaged F1-score of approximately 0.85. The classification report Table 8 and confusion matrix (Figure 10) show consistent but limited improvement. The training curves (Figures 11, 12) indicate stable optimization behavior.

These results indicate that post-hoc adaptation can recover additional discriminative signal. However, the relatively small gain suggests that the feature space produced by group-shuffle alone is not sufficiently expressive to fully benefit from post-hoc learning (Table 8).

**Table 8:** Baseline model + group-shuffle + R-Head performance classification report.

Class	Precision	Recall	F1-score	Support
0	0.9370	0.9563	0.9465	1,601
1	0.7977	0.7282	0.7614	379
Accuracy		0.9126		1,980
Macro avg	0.8673	0.8423	0.8540	1,980
Weighted avg	0.9103	0.9126	0.9111	1,980

#### 4.3.3 Experiment 3: baseline + GSSE + redundant head

##### 4.3.3.1 GSSE-enhanced base model

In the third experiment, the DenseNet-121 backbone is augmented with the proposed Group–Squeeze-Excitation–Shuffle (GSSE) block after the final dense block. This design targets feature redundancy, channel-wise noise, and limited cross-channel interaction.

**Figure 13:**
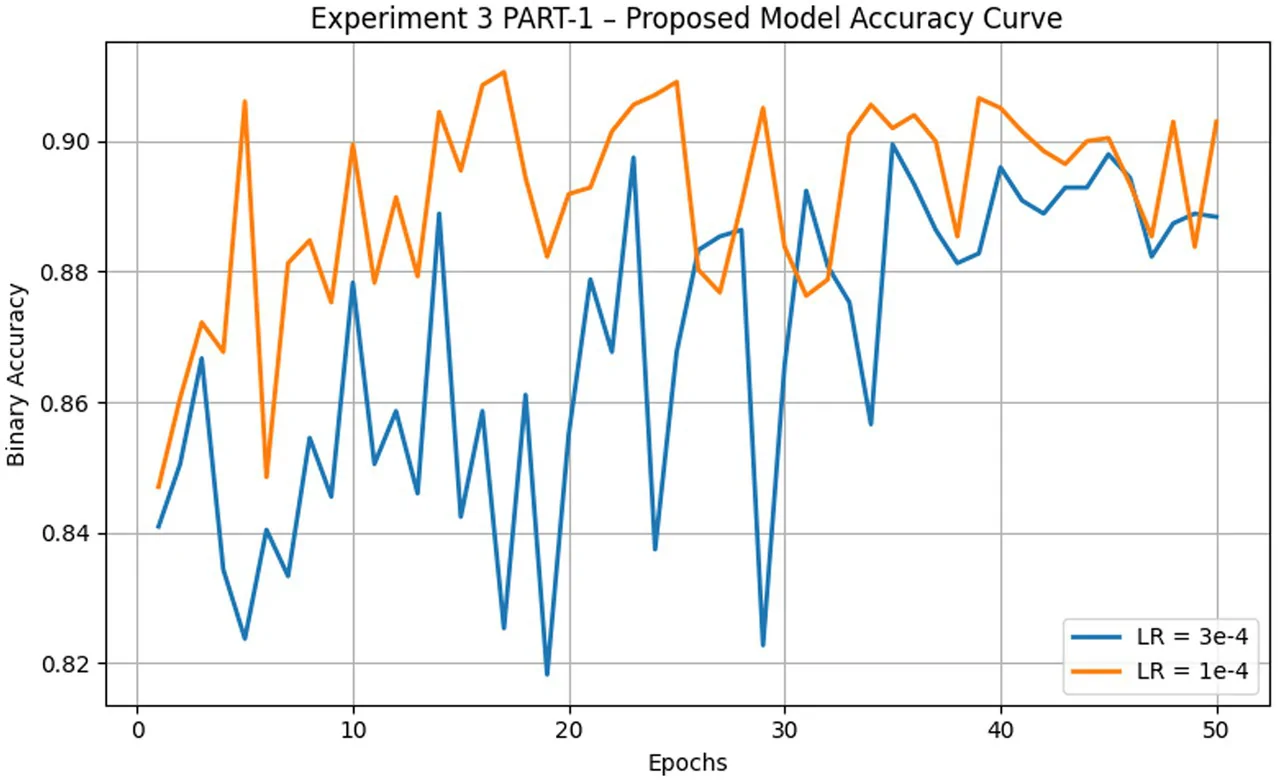
Baseline model + GSSE training-tuning binary accuracy curve.

**Figure 14:**
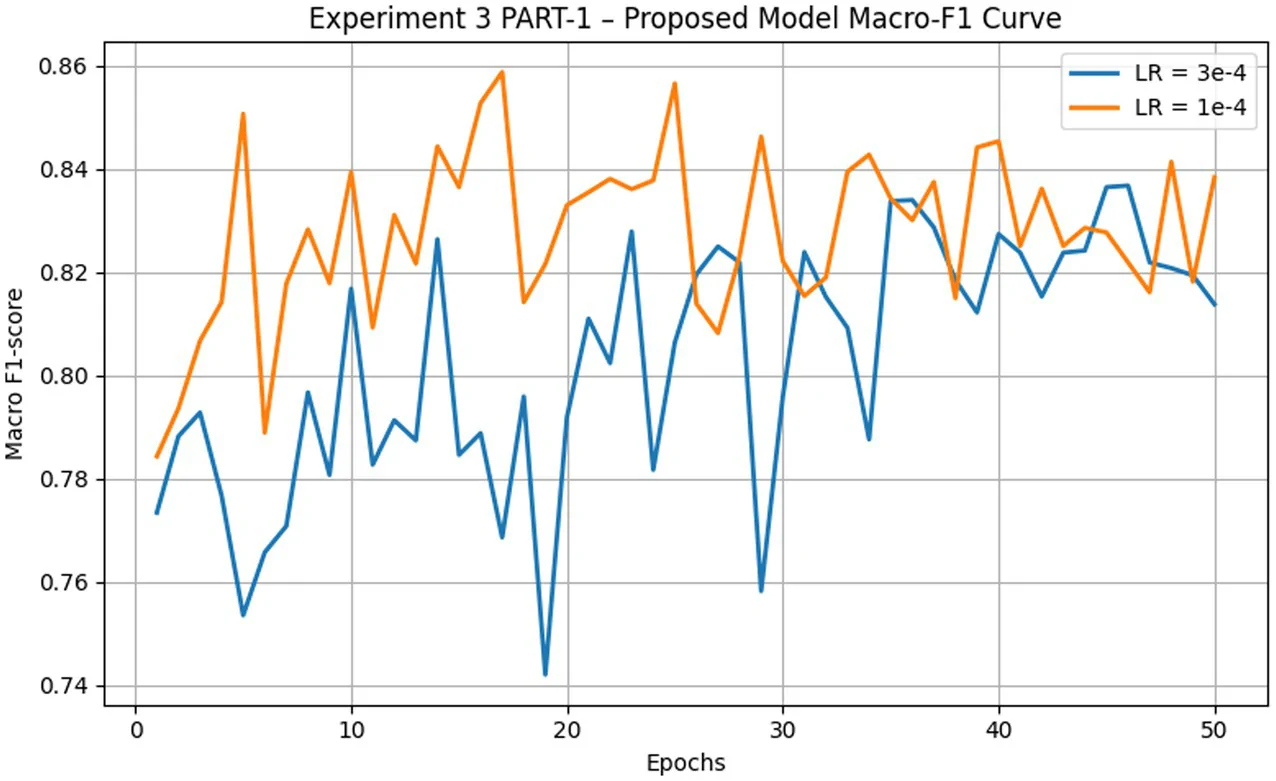
Baseline model + GSSE training-tuning macro avg F1 curve.

Training follows the same protocol as previous experiments. The best GSSE-enhanced model achieves a binary accuracy of 91.06% and a macro-averaged F1-score of 0.8588. As shown in Table 9 and the training curves (Figures 13, 14), the model converges stably with performance comparable to the baseline.

Despite introducing a more expressive feature representation, the GSSE-enhanced model does not improve performance under end-to-end training. This indicates that increased feature richness does not necessarily translate to improved classification when the primary head is jointly optimized. The result highlights a mismatch between feature capacity and classifier adaptation, motivating the use of post-hoc learning (Table 9).

**Table 9:** Baseline model + GSSE performance classification report.

Class	Precision	Recall	F1-score	Support
0	0.9512	0.9375	0.9443	1,601
1	0.7512	0.7968	0.7734	379
Accuracy		0.9106		1,980
Macro avg	0.8512	0.8672	0.8588	1,980
Weighted avg	0.9129	0.9106	0.9116	1,980

##### 4.3.3.2 Redundant head on GSSE backbone

A redundant head (R-Head) is trained on the frozen GSSE-enhanced backbone. Both the backbone and original classification head are frozen to preserve representational stability. The redundant head is trained using frozen feature representations together with frozen logits, which provide class-aware priors during optimization.

**Figure 15:**
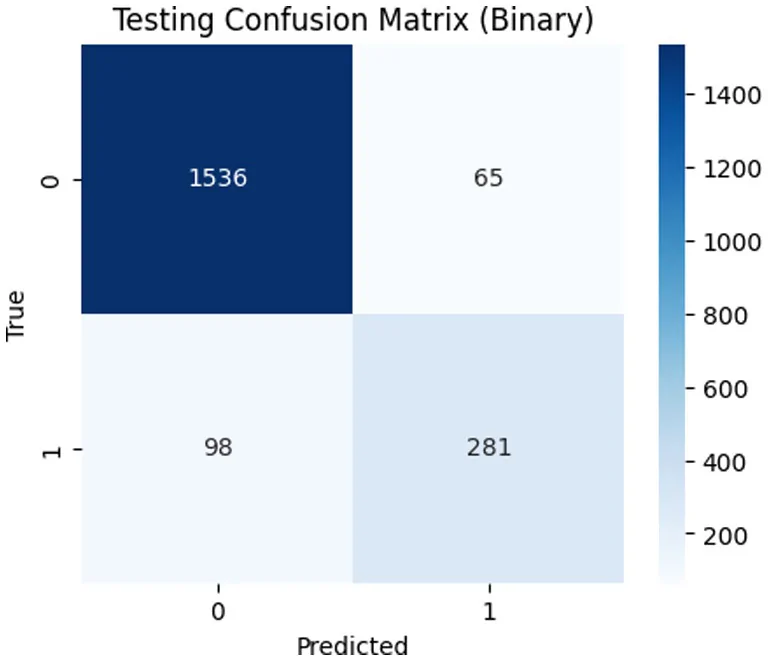
Baseline model + GSSE + R-Head1 performance confusion matrix.

**Figure 16:**
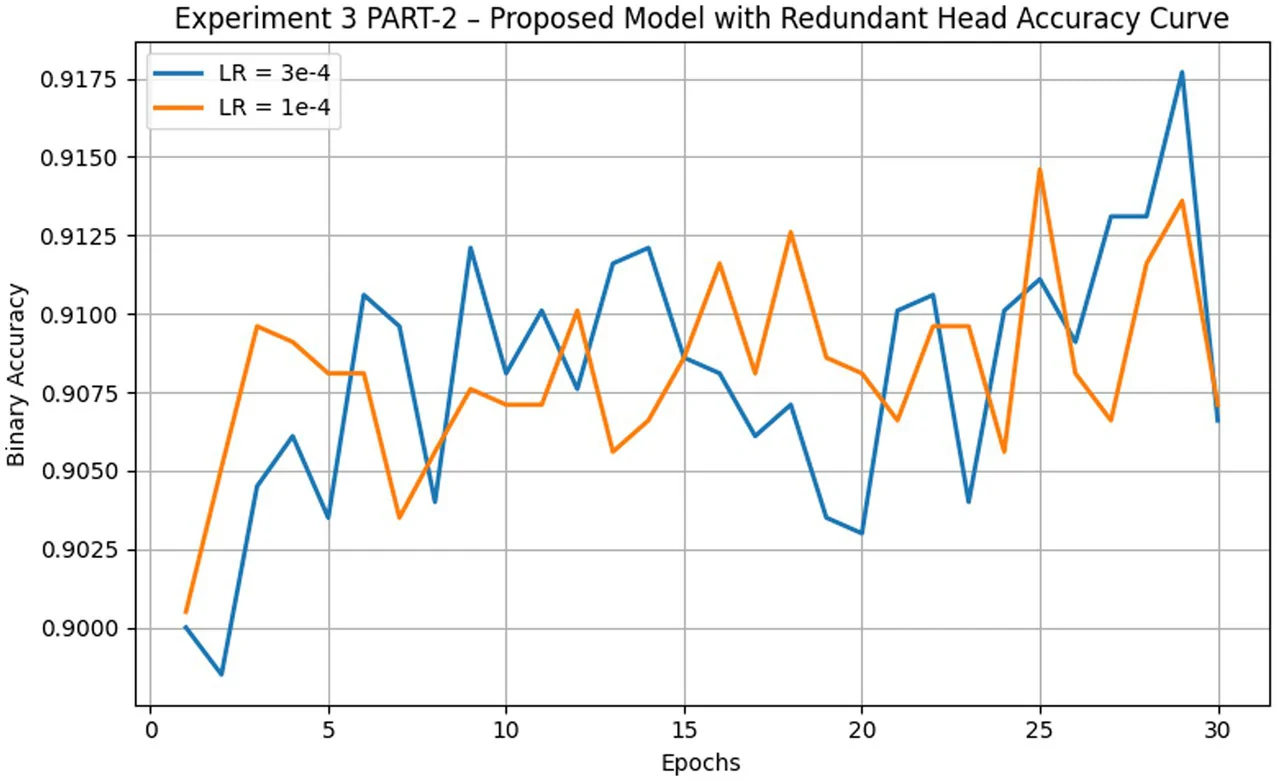
Baseline Model + GSSE + R-Head1 training-tuning binary accuracy curve.

**Figure 17:**
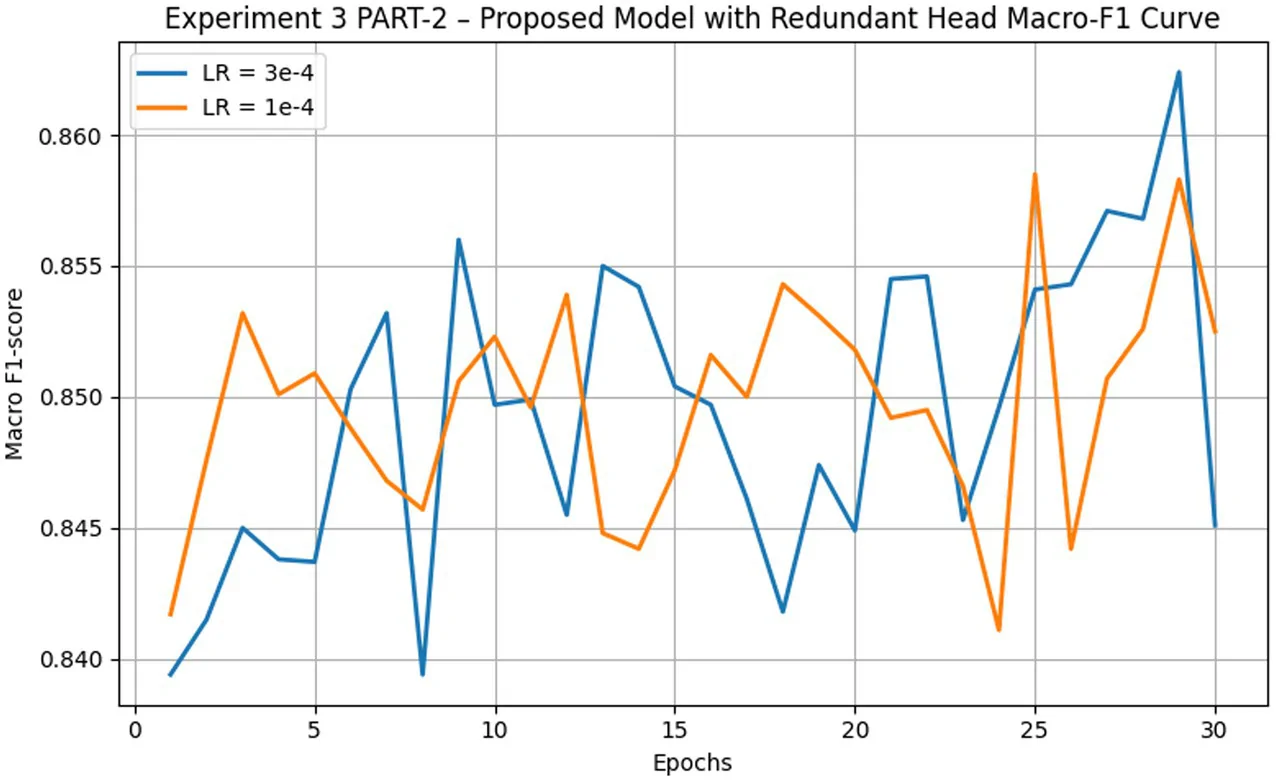
Baseline model + GSSE + R-Head1 training-tuning macro avg F1 curve.

Training is conducted for up to 30 epochs with learning rates {1 × 10^−4^, 3 × 10^−4^}. The best configuration achieves a binary accuracy of 91.77% and a macro-averaged F1-score of 0.8624. The classification report Table 10 and confusion matrix (Figure 15) show improved discrimination compared to the GSSE base model, while the training curves (Figures 16, 17) indicate stable convergence.

This improvement demonstrates that GSSE introduces latent discriminative information that is not effectively utilized by the primary head but can be recovered through post-hoc adaptation (Table 10).

**Table 10:** Baseline model + GSSE + R-Head1 performance classification report.

Class	Precision	Recall	F1-score	Support
0	0.9400	0.9594	0.9496	1,601
1	0.8121	0.7414	0.7752	379
Accuracy		0.9177		1,980
Macro avg	0.8761	0.8504	0.8624	1,980
Weighted avg	0.9155	0.9177	0.9162	1,980

##### 4.3.3.3 Enhanced redundant head tuning (GSARC)

To further explore adaptation capacity, learning rates are extended to {1 × 10^−3^, 3 × 10^−3^} while maintaining the frozen-backbone setup. The redundant head is trained under the same protocol.

This configuration achieves a binary accuracy of 92.42% and a macro-averaged F1-score of 0.8748, representing the best performance across all experiments. Compared to the GSSE base model, this corresponds to an improvement of approximately 1.4% in accuracy and 1.6% in macro-F1, while also surpassing the strongest baseline.

To assess robustness, experiments are repeated with random seeds 123 and 999. The redundant head consistently improves performance, yielding a mean gain of 0.97% (SD 0.38), confirming that the improvement is stable and not attributable to random initialization.

#### 4.3.4 Robustness across random seeds and statistical analysis

To evaluate the consistency of the proposed GSARC framework, experiments were repeated using three different random seeds. Table 11 presents the classification accuracies achieved by the GSSE baseline and the proposed GSARC framework across all evaluated seeds.

**Table 11:** Accuracy comparison between GSSE and GSARC across different random seeds.

Random seed	GSSE accuracy (%)	GSARC accuracy (%)	Improvement (percentage points)
42	91.06	92.42	+1.36
123	89.83	90.93	+1.10
999	90.14	90.59	+0.45
Mean	90.34	91.31	+0.97

Across all evaluated seeds, GSARC consistently outperformed the GSSE baseline, yielding accuracy improvements of 1.36, 1.10, and 0.45 percentage points for seeds 42, 123, and 999, respectively. The mean improvement across all runs was 0.97 percentage points, indicating that the proposed post-hoc adaptation strategy provides a consistent performance benefit regardless of initialization conditions.

To further quantify the reliability of the observed gains, additional statistical analysis was performed. Table 12 summarizes the results of the statistical evaluation.

**Table 12:** Statistical evaluation of GSARC versus GSSE across multiple random seeds.

Metric	Value
Number of seeds	3
Mean accuracy gain	0.97 percentage points
Standard deviation	0.47 percentage points
95% confidence interval	[−0.20, 2.14] percentage points
Paired *t*-statistic	3.58
Degrees of freedom	2
*p*-value (two-tailed)	0.070

The observed gains exhibited a standard deviation of 0.47 percentage points, indicating relatively low variability across runs. The corresponding 95% confidence interval for the improvement was [−0.20, 2.14] percentage points. A paired *t*-test comparing GSSE and GSARC accuracies yielded t(2) = 3.58 with a two-tailed *p*-value of 0.070.

Although the limited number of runs prevents strong statistical significance claims at the conventional *α* = 0.05 threshold, the proposed GSARC framework achieved positive improvements for every evaluated seed. This consistent behavior suggests that the observed gains are repeatable and are not solely attributable to random initialization effects. Collectively, these findings support the robustness of the proposed lightweight post-hoc adaptation mechanism while maintaining its computational efficiency and deployment simplicity.

**Figure 18:**
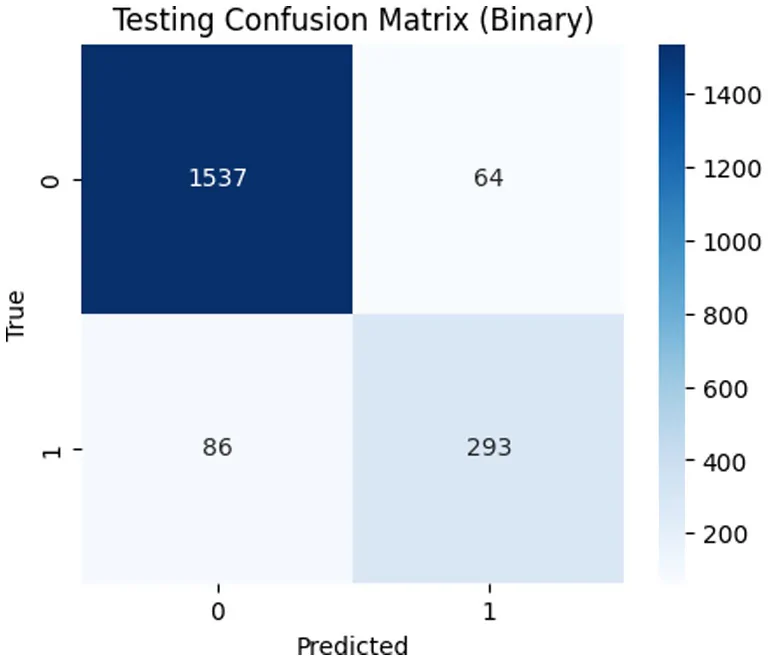
Baseline model + GSSE + R-Head2 performance confusion matrix.

**Figure 19:**
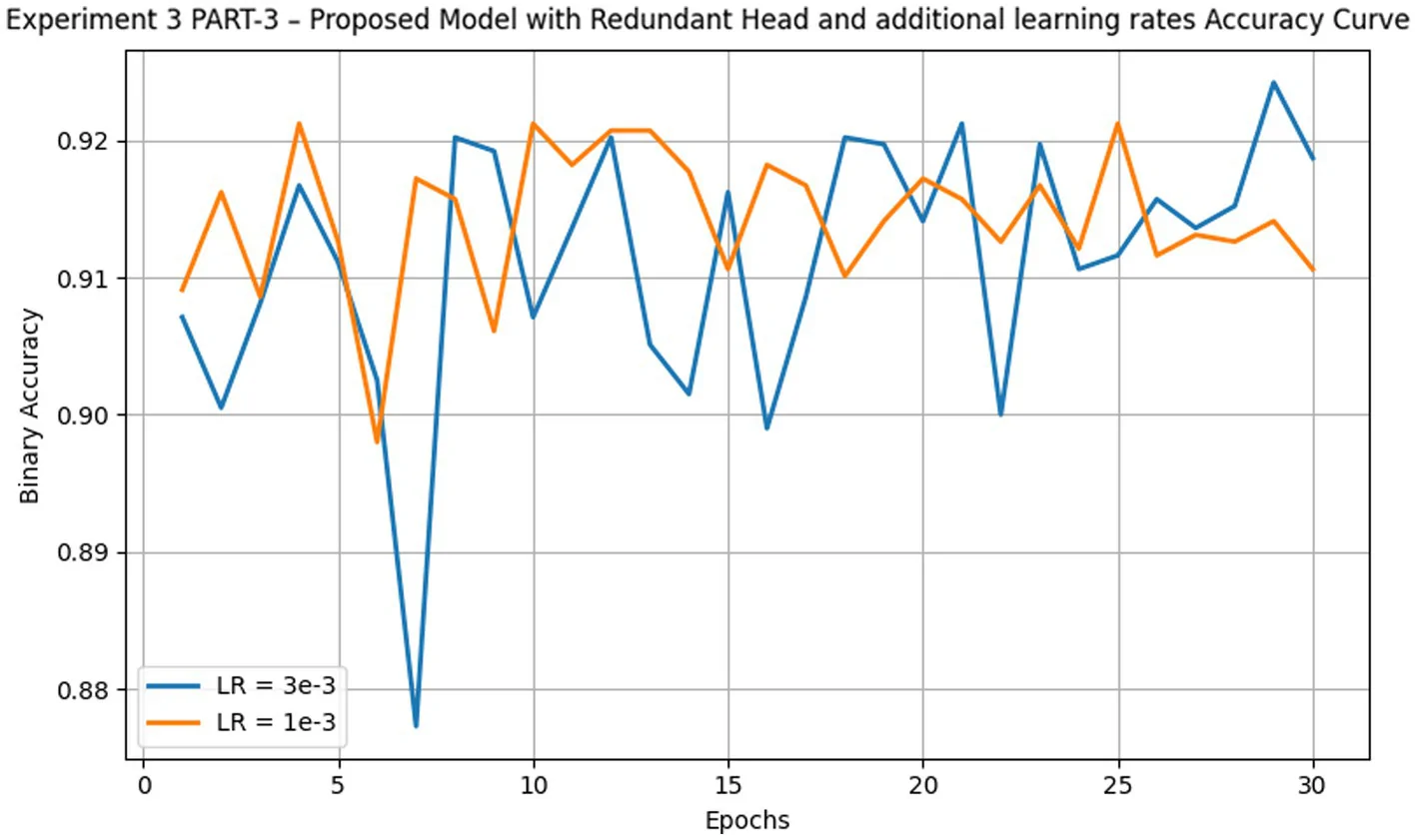
Baseline Model + GSSE + R-Head2 training-tuning binary accuracy curve.

**Figure 20:**
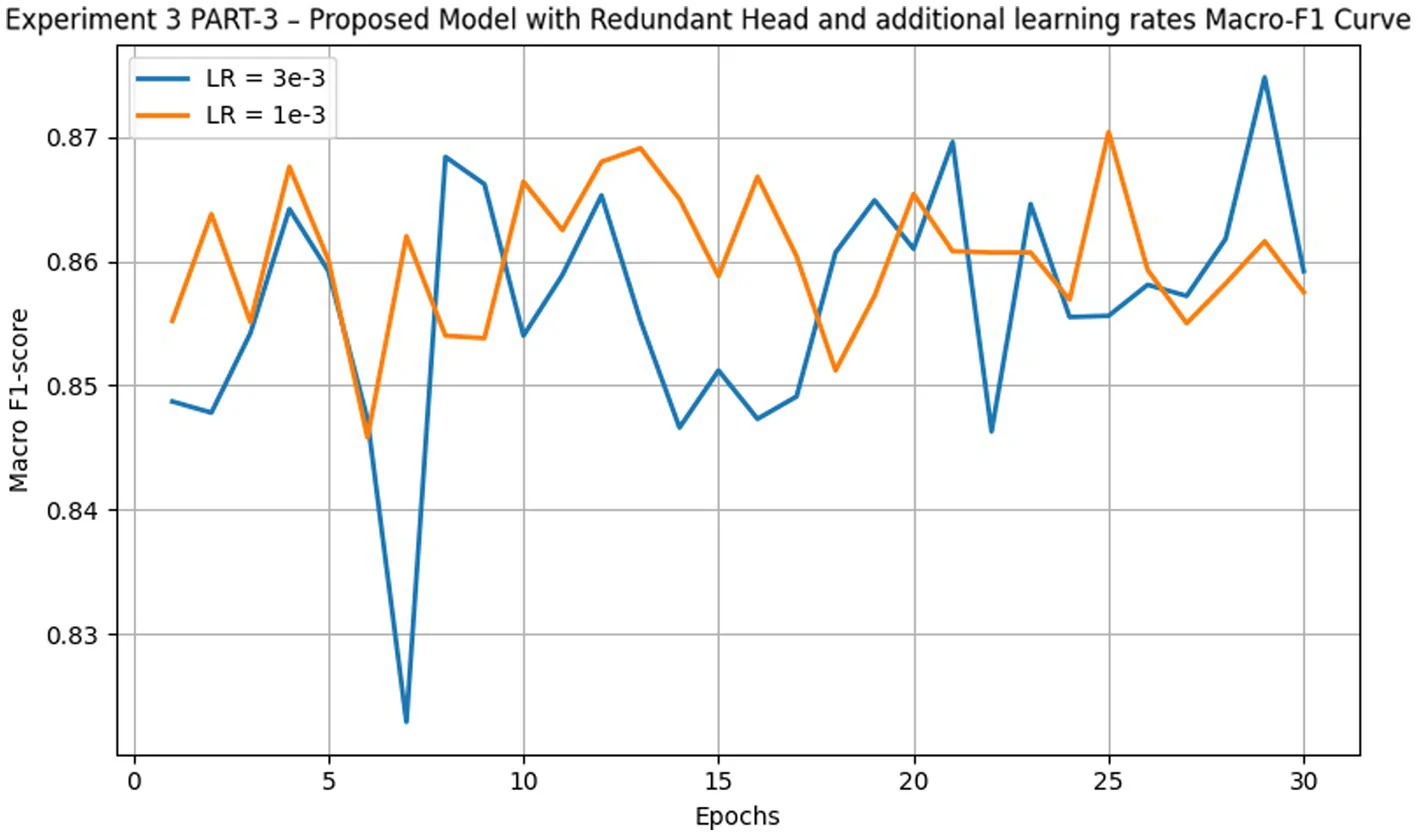
Baseline model + GSSE + R-Head2 training-tuning macro avg F1 curve.

The classification report Table 13 and confusion matrix (Figure 18) show improved class-wise balance, while the training curves (Figures 19, 20) demonstrate faster convergence. This suggests that most discriminative information is already present in the frozen feature space and can be efficiently extracted through lightweight post-hoc optimization (Table 13).

**Table 13:** Baseline model + GSSE + R-Head2 performance classification report.

Class	Precision	Recall	F1-score	Support
0	0.9470	0.9600	0.9535	1,601
1	0.8207	0.7731	0.7962	379
Accuracy		0.9242		1,980
Macro avg	0.8839	0.8666	0.8748	1,980
Weighted avg	0.9228	0.9242	0.9234	1,980

##### 4.3.4.1 Comparative analysis with existing methods

Table 14 compares the proposed GSARC model with representative approaches for skin lesion classification. Earlier custom CNN models, such as those by Bechelli et al. and Mehr et al., report accuracies between 82 and 89.3%. Transfer learning approaches improve performance, with Ameri A. and Salmaa et al. achieving 84 and 91.3%, respectively. Roge et al. report 92% using ensemble-based methods with increased computational complexity.

**Table 14:** Comparison of proposed model with existing works.

Source	Method	Accuracy
Bechelli and Delhommelle (2022)	Custom CNN	82
Ameri (2020)	Transfer learning	84
Mehr and Ameri (2022)	Custom CNN	89.3
Salamaa and Aly (2021)	Transfer learning	91.3
Roge et al. (2023)	Transfer learning	92
Proposed model	GSARC model	92.4

The proposed GSARC model achieves a binary accuracy of 92.4%, outperforming prior single-model approaches while avoiding ensemble construction or backbone retraining. Unlike existing methods that improve performance through architectural scaling or model aggregation, the proposed approach enhances feature utilization through lightweight post-hoc adaptation.

The comparisons presented in Table 14 should be interpreted as indirect comparisons because the referenced studies employ different datasets, preprocessing procedures, train-test partitions, augmentation strategies, and evaluation settings. Consequently, the reported results are intended to provide contextual reference rather than a controlled head-to-head benchmark. Despite these differences, the proposed GSARC framework demonstrates competitive performance while maintaining a lightweight post-hoc adaptation strategy that avoids backbone retraining and ensemble construction.

### 4.4 Multi-class evaluation

While binary malignant-versus-benign classification reflects the primary clinical screening objective, evaluating the model under the original seven-class formulation provides additional insight into class-specific discrimination performance and the ability of the proposed framework to distinguish among visually similar lesion categories (Table 15).

**Table 15:** Multiclass performance comparison between GSSE and GSARC.

Model	7-class accuracy (%)	Macro F1 (%)
GSSE baseline	86.21	74.96
GSARC (proposed)	86.52	77.14
Improvement	+0.30	+2.19

**Figure 21:**
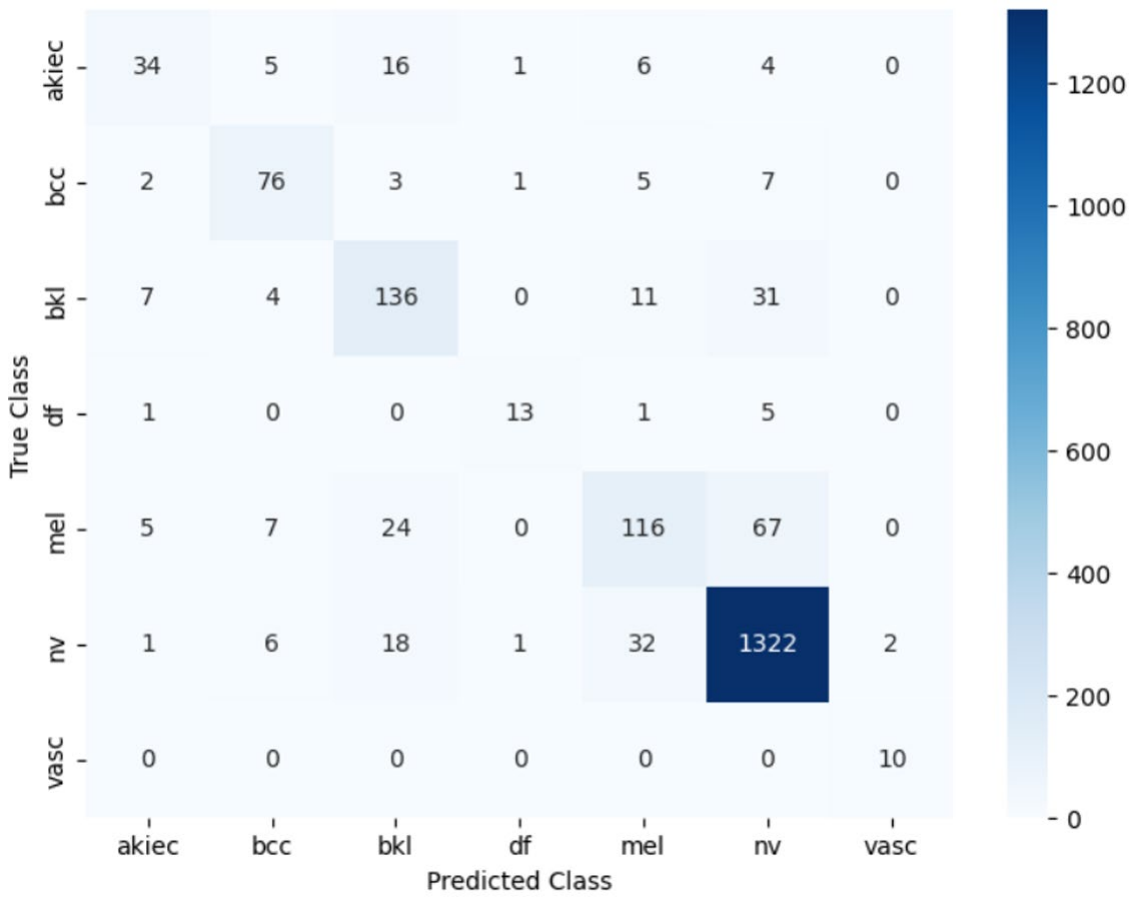
Seven-class confusion matrix of the proposed GSARC framework.

To further assess the effectiveness of the proposed GSARC framework, a comprehensive seven-class evaluation was conducted on the lesion-wise test set. Table 16 summarizes the multiclass performance metrics, including overall accuracy, macro-averaged F1-score, weighted F1-score, and class-wise precision, recall, and F1-score values. In addition, the multiclass confusion matrix is presented in Figure 21 to visualize inter-class prediction patterns.

**Table 16:** Class-wise performance of the proposed GSARC framework.

Class	Precision	Recall	F1-Score	Support
akiec	0.6800	0.5152	0.5862	66
bcc	0.7755	0.8085	0.7917	94
bkl	0.6904	0.7196	0.7047	189
df	0.8125	0.6500	0.7222	20
mel	0.6784	0.5297	0.5949	219
nv	0.9206	0.9566	0.9383	1,382
vasc	0.8333	1.0000	0.9091	10
Macro average	0.7701	0.7399	0.7496	1,980
Weighted average	0.8554	0.8621	0.8570	1,980

The proposed GSARC framework achieved a seven-class classification accuracy of 86.52% and a macro-averaged F1-score of 77.14%. Compared with the GSSE-enhanced baseline, GSARC improved overall accuracy from 86.21 to 86.52% (+0.30 percentage points) while increasing macro-F1 from 74.96 to 77.14% (+2.19 percentage points). Although the improvement in overall accuracy is modest, the substantially larger gain in macro-F1 demonstrates improved class balance and more effective discrimination across minority lesion categories.

Analysis of the class-wise performance reveals strong classification capability across multiple lesion types. High performance is observed for melanocytic nevi (nv), which achieved an F1-score exceeding 0.93, while vascular lesions (vasc) maintained excellent performance despite limited sample availability. The results further indicate improved discrimination among diagnostically challenging lesion categories, including basal cell carcinoma (bcc), benign keratosis-like lesions (bkl), and melanoma (mel), suggesting that the redundant post-hoc adaptation mechanism successfully exploits residual discriminative information present within the frozen feature space.

The confusion matrix further illustrates that the majority of predictions remain concentrated along the diagonal, indicating strong class-specific recognition. The remaining misclassifications primarily occur among lesion categories with known visual similarity, particularly melanoma, benign keratosis-like lesions, and melanocytic nevi. Such confusion patterns are consistent with the intrinsic difficulty of dermoscopic lesion classification and reflect challenges commonly reported in the literature.

The concentration of predictions along the diagonal indicates strong class discrimination, while off-diagonal entries highlight remaining confusion among visually similar lesion categories.

These findings support the central hypothesis of this work: enriched feature representations contain additional class-specific information that may not be fully utilized by the jointly optimized primary classification head. By introducing a lightweight post-hoc redundant head operating on frozen features and logits, GSARC is able to recover part of this residual discriminative capacity without modifying the backbone network.

Overall, the multiclass evaluation demonstrates that the benefits of the proposed GSARC framework extend beyond clinically aggregated binary labels and contribute to improved fine-grained lesion discrimination under the original seven-class formulation.

It is important to note that the multiclass configuration was not subjected to extensive architecture-specific optimization, as the primary objective of this work was to preserve the lightweight, computationally efficient, and deployment-friendly nature of the proposed post-hoc adaptation framework. Consequently, additional gains may be achievable through dedicated multiclass hyperparameter tuning, more expressive redundant-head architectures, or architecture-specific optimization strategies. However, such modifications would introduce additional complexity and fall outside the scope of the lightweight post-training adaptation objective considered in this study.

## 5 Limitations

The proposed method presents several limitations that should be taken into account when interpreting the findings.

First, although evaluation was conducted on both HAM10000 and ISIC-2019, the experiments remain limited to dermoscopic skin lesion datasets. Additional validation on external clinical datasets and other medical imaging domains would further strengthen conclusions regarding the generalizability of the proposed post-hoc adaptation mechanism.

Second, while the proposed framework was evaluated using both DenseNet-121 and EfficientNet-B0 backbones, its effectiveness on other architectural paradigms, particularly transformer-based vision models, remains unexplored. Investigating the interaction between post-hoc adaptation and transformer-derived feature representations represents an important direction for future research.

Third, while training is conducted using a seven-class formulation, performance assessment is limited to binary classification. Although this reflects clinical relevance, it may conceal detailed class-wise performance variations.

Fourth, the redundant head is designed as a shallow linear classifier. More complex post-hoc variants, including deeper or non-linear architectures, are not examined in this work.

Finally, the post-hoc adaptation procedure is carried out in an offline manner. The approach is not designed for real-time inference or continual learning settings, which may be important in practical deployment scenarios.

## 6 Generalization evaluation on an independent dataset: ISIC-2019

To examine the generalization ability of the proposed redundant head framework beyond the HAM10000 setting, additional experiments were conducted using the ISIC-2019 skin lesion dataset. This dataset consists of 25,331 dermoscopic images spanning eight diagnostic categories (AK, BCC, BKL, DF, MEL, NV, SCC, VASC).

To maintain consistency with the primary experimental protocol, a seven-class training setup was used by excluding the VASC category. For evaluation, binary malignancy labels were constructed, where AK, BCC, MEL, and SCC were grouped as malignant, and BKL, DF, and NV were grouped as benign.

An 80/20 stratified split based on the seven-class labels was applied, yielding 20,264 training samples and 5,067 test samples. The class distribution was preserved across splits: the training set includes 12,792 benign and 7,472 malignant samples, while the test set contains 3,199 benign and 1,868 malignant samples. All experiments were conducted with deterministic seed control (seed = 42) to ensure reproducibility.

To further assess the architecture-agnostic behavior of the proposed method and evaluate its performance on a strong modern baseline, EfficientNet-B0 was selected for this set of experiments.

### 6.1 Baseline: EfficientNet-B0

An EfficientNet-B0 model pretrained on ImageNet was fine-tuned using a seven-class classification objective with cross-entropy loss. During evaluation, predictions were mapped from the seven-class output space into binary malignant and benign categories. The model was trained for 30 epochs using a learning rate of 1 × 10^−4^, and the best checkpoint was chosen based on binary validation accuracy. This baseline reached a binary accuracy of 90.37%, reflecting strong representational capability and effective adaptation to the malignancy classification task.

### 6.2 Redundant head on frozen EfficientNet

The backbone was subsequently frozen, and the redundant head mechanism was applied. Global pooled feature vectors were obtained and concatenated with the original seven-class logits. A lightweight linear classifier was then trained on this combined representation.

To enhance computational efficiency, the backbone features and logits were cached and reused throughout training. This approach avoided repeated forward passes and enabled efficient experimentation with different redundant head configurations.

### 6.3 Learning rate exploration

A wide range of learning rates was explored to assess the adaptability of the redundant head. The evaluated values included 4 × 10^−3^, 2 × 10^−3^, 7 × 10^−3^, 4 × 10^−2^, and 7 × 10^−2^. Each configuration was trained for 150 epochs, with performance monitored at every epoch. The final selection was based on macro-averaged F1-score.

### 6.4 Best configuration

The best-performing setup was obtained with a learning rate of 4 × 10^−2^. The top result was observed at epoch 3, achieving a binary accuracy of 90.84%.

### 6.5 Performance gain over baseline

Relative to the frozen EfficientNet-B0 baseline, the redundant head produces a small but consistent improvement in binary accuracy (90.84% vs. 90.37%). This gain is smaller than that observed in the HAM10000 experiments, which is expected since EfficientNet-B0 already demonstrates strong adaptation during fine-tuning.

Nevertheless, the method continues to extract additional discriminative information from the augmented feature representation. The improvement appears early in training, remains stable across a wide range of learning rates, and does not require any modification of the backbone parameters.

### 6.6 Interpretation

The results reveal several consistent patterns of behavior. When the baseline classifier is already well optimized, the redundant head delivers modest yet consistent improvements. In contrast, larger gains are observed when the baseline head does not fully exploit the feature space, as demonstrated in the DenseNet-121 experiments on HAM10000.

Importantly, the method does not negatively impact performance even when applied to strong baselines and remains computationally efficient due to backbone freezing and feature caching. These observations support the view that the proposed approach serves as a stability-preserving mechanism for improved feature utilization, rather than replacing backbone training.

Overall, the results indicate that lightweight post-hoc adaptation can recover residual discriminative information from learned feature representations, leading to performance improvements without increasing training complexity.

The EfficientNet-B0 experiments further suggest that the proposed framework is not inherently tied to a specific convolutional architecture. Although the magnitude of improvement varies depending on the baseline model’s level of feature utilization, the redundant head consistently recovers additional discriminative information without requiring modification of backbone parameters. This observation indicates that the proposed post-hoc adaptation strategy may be broadly applicable across different convolutional architectures.

## 7 Conclusion

This study presents GSARC, a lightweight post-hoc redundant head framework designed to improve feature-space adaptation after model training without modifying or retraining the backbone network. The approach integrates feature-space enrichment through the GSSE block with a logit-aware redundant head trained on frozen representations. This formulation enables the recovery of previously underutilized discriminative information while maintaining the stability of the learned feature extractor. Experimental results on the HAM10000 dataset show that feature enhancement alone does not necessarily translate into improved classification performance. However, the proposed post-hoc redundant head effectively utilizes the enriched feature space. The final GSARC configuration achieves a binary classification accuracy of 92.42% with a macro-averaged F1-score of 0.8748, outperforming the baseline DenseNet-121 model while introducing only minimal computational overhead.

To evaluate generalization, additional experiments were conducted on the ISIC-2019 dataset using an EfficientNet-B0 backbone. Even under a strong baseline, the redundant head consistently captures additional discriminative signal, improving binary accuracy from 90.37 to 90.84% without requiring backbone retraining. These results indicate that the proposed method functions as a stable post-hoc enhancement mechanism that improves the utilization of learned feature representations.

Additional multiclass evaluation further demonstrates that the proposed framework improves fine-grained lesion discrimination under the original seven-class formulation, indicating that the benefits of post-hoc adaptation extend beyond binary malignant-versus-benign classification.

Although the proposed framework demonstrates promising performance on benchmark skin-lesion datasets, the reported results should be interpreted as evidence of technical feasibility rather than immediate clinical readiness. Additional validation on external datasets, prospective evaluation studies, and broader clinical assessment are necessary before deployment in real-world diagnostic workflows. Consequently, the proposed framework is best viewed as a clinical decision-support tool intended to assist, rather than replace, expert medical judgment.

Overall, the findings demonstrate that lightweight post-training adaptation can yield measurable performance improvements in medical image classification systems while preserving computational efficiency and deployment flexibility.

## 8 Future work

Several directions can be explored to further extend this work. Evaluating the proposed method across additional dermoscopic datasets would provide a more comprehensive understanding of its robustness and generalization capability. Extending the approach to other backbone architectures, including both convolutional and transformer-based models, would help assess its broader applicability.

Future studies may also investigate the effectiveness of the redundant head under full multi-class evaluation settings, beyond binary classification. In addition, exploring more expressive post-hoc head designs, such as deeper or regularized architectures, could further enhance feature utilization.

Another important direction involves extending the framework toward online and continual learning settings. While the current implementation performs offline post-hoc adaptation after backbone training, future work could investigate incremental redundant-head updates that accommodate evolving data distributions without modifying the underlying feature extractor. Since the proposed framework benefits from learning on stable frozen representations, continuously changing feature spaces may introduce representational drift and reduce the effectiveness of post-hoc adaptation. Consequently, future work may explore hybrid adaptation strategies that periodically incorporate newly observed data while preserving feature-space stability. Such extensions could enable adaptation in dynamic clinical environments while maintaining the computational efficiency, deployment flexibility, and regulatory advantages associated with frozen backbone representations. Evaluating the method in real-world clinical environments, particularly under constraints related to latency and computational resources, would also be valuable.

Finally, conducting systematic comparisons with recent state-of-the-art skin lesion classification methods under standardized training and evaluation protocols would provide deeper insight into the relative performance and efficiency of the proposed approach.

## Data Availability

Publicly available datasets were analyzed in this study. This data can be found here: https://www.kaggle.com/datasets/kmader/skin-cancer-mnist-ham10000.
